# Pharmacological characterization and preclinical evaluation of 11h: a novel, brain-penetrant PDE4 inhibitor for neurological disorders

**DOI:** 10.3389/fphar.2025.1720327

**Published:** 2026-01-08

**Authors:** Jacob Lackovic, Sougata Dey, Sara Jane Ward, Nigel H. Greig, David Tweedie, Nassim Beiranvand, Uksha Saini, Dev Chatterjee, Atul Varadhachary

**Affiliations:** 1 Fannin Partners LLC, Houston, TX, United States; 2 Center for Substance Abuse Research, Department of Neural Sciences, Temple University, Philadelphia, PA, United States; 3 Drug Design & Development Section, Translational Gerontology Branch, Intramural Research Program National Institute on Aging, NIH, Baltimore, MD, United States; 4 Accelerator for Cancer Therapeutics, TMC Innovation, Texas Medical Center, Houston, TX, United States; 5 Goldenrod Therapeutics, Inc., Houston, TX, United States

**Keywords:** cAMP, microglia, MyD88, neurodegeneration, neuroimmune, neuroinflammation, neurotherapeutics, PDE4

## Abstract

**Background:**

Phosphodiesterase 4 (PDE4) inhibitors hold promise for treating neuroinflammatory and neurodegenerative disorders, but their clinical application in central nervous system (CNS) diseases has been limited by insufficient brain penetration and adverse effects, especially nausea and vomiting. In this study, we characterize compound 11h, a novel, orally available, brain-penetrant PDE4 inhibitor designed to address these limitations.

**Methods:**

11h was evaluated using integrated computational, in vitro, and in vivo approaches, including Schrödinger-based molecular docking against human PDE4A10, luminescence-based cAMP assays for PDE4 isoform inhibition, and inflammatory assays in murine macrophages, microglia-like cells, and human PBMCs. In vivo efficacy, pharmacokinetics, brain and tissue distribution, tolerability, emesis liability, and behavioral effects were assessed across mouse, rat, and ferret models, alongside comprehensive safety pharmacology encompassing CYP450 inhibition, cardiac ion channel profiling, mutagenicity, and off-target screening.

**Results:**

11h demonstrated potent, broad-spectrum inhibition across all four PDE4 isoforms, including key PDE4D splice variants (PDE4D2, PDE4D3), with low nanomolar IC50 values. *In vitro*, 11h significantly reduced the release of tumor necrosis factor alpha (TNFα), interleukin 6 (IL-6), and nitrites in lipopolysaccharide (LPS)-stimulated macrophages, microglia-like cells, and human peripheral blood mononuclear cells without affecting cell viability. *In vivo*, 11h attenuated neuroinflammation in LPS-treated mice by decreasing M1 macrophages and CD4+ T cells, increasing M2 macrophages, and downregulating pro-inflammatory cytokines and MyD88 pathway genes. Pharmacokinetic analysis in rats confirmed strong oral bioavailability, dose-proportional systemic exposure, and sustained brain concentrations exceeding plasma levels for up to 48 h post-dose. Importantly, 11h did not induce vomiting in the ferret emesis model even at doses exceeding 50-fold the efficacious levels in rodent disease models, and was well tolerated in behavioral assays, where it produced anxiolytic-and antidepressant-like effects. Safety profiling revealed no cytotoxicity, genotoxicity, or significant inhibition of cardiac ion channels or cytochrome P450 enzymes. Consistent with this experimental profile, molecular docking suggested that 11h preferentially engages a pocket within the PDE4 catalytic domain, with high predicted binding affinity and ligand efficiency driven by hydrophobic and electrostatic interactions.

**Conclusion:**

These findings suggest that 11h provides broad PDE4 inhibition with a favorable tolerability and pharmacokinetic profile relative to approved PDE4 inhibitors, supporting its further development as a therapeutic candidate for CNS disorders characterized by neuroinflammation.

## Introduction

Over the past decade, phosphodiesterase 4 (PDE4) enzymes have become compelling therapeutic targets for central nervous system (CNS) disorders. PDE4 enzymes are key regulators of intracellular cyclic adenosine monophosphate (cAMP) signaling, a critical second messenger involved in numerous cellular processes, including gene transcription, synaptic plasticity, cell survival, and inflammatory responses (Conti et al., 2003; Blokland et al., 2019a). Within the CNS, PDE4 isoforms, particularly PDE4A, PDE4B, and PDE4D, are broadly expressed within neurons, astrocytes, microglia, and oligodendrocytes, where they control localized cAMP pools and mediate the downstream activation of protein kinase A (PKA), cAMP response element-binding protein (CREB), and other effectors (Blokland et al., 2019a; Pérez-Torres et al., 2000; Lakics et al., 2010). The spatial and temporal regulation of cAMP by PDE4 is essential for maintaining neuroplasticity, modulating neuroimmune interactions, and enabling proper neuronal adaptation to external stimuli (Blokland et al., 2019a; Donders et al., 2024; Rombaut et al., 2021).

Dysregulated PDE4 activity has been strongly implicated in a broad range of neurological and neuropsychiatric disorders. In neurons, excessive PDE4-mediated cAMP degradation impairs CREB-dependent transcription, disrupts synaptic plasticity, and reduces the brain’s capacity for functional remodeling throughout the central executive network, contributing to cognitive decline and mood dysregulation (Kandel, 2012; Janes et al., 2009; Bollen et al., 2014). In glial cells, particularly microglia and astrocytes, aberrant PDE4 activity promotes chronic neuroinflammation by enhancing the production of pro-inflammatory cytokines, such as Tumor Necrosis Factor alpha (TNFα) and interleukin-6 (IL-6), and maintaining an activated immune state (Zhang et al., 2022; Jin et al., 2005; Jin and Conti, 2002). These cellular effects have been linked to pathogenesis across Alzheimer’s (AD) and Parkinson’s disease, multiple sclerosis (MS), substance use disorders, and schizophrenia, among others (Blokland et al., 2019a; Chen et al., 2009; Schepers et al., 2023; Singh, 2022).

Preclinical studies have consistently demonstrated the therapeutic potential of PDE4 inhibition across diverse CNS disease models, highlighting its broad pharmacological activity. For example, in rodent models of depression, PDE4 inhibitors such as rolipram produce antidepressant-like effects comparable to selective serotonin reuptake inhibitors (SSRIs), driven by enhanced cAMP signaling and subsequent upregulation of CREB-mediated transcription of neurotrophic factors (Cong et al., 2023; Wachtel and Schneider, 1986; Zhang et al., 2017). In disease models associated with pronounced neuroinflammation, such as experimental autoimmune encephalomyelitis and traumatic brain injury, PDE4 inhibition attenuates glial activation, reduces pro-inflammatory cytokine production, and promotes neuroprotection (Schepers et al., 2023; Titus et al., 2016). Similarly, in models of cognitive impairment, PDE4 blockade improves hippocampal long-term potentiation and enhances memory performance in behavioral tasks (Cong et al., 2023; Gong et al., 2004; Imanishi et al., 1997). Clinical trials have largely echoed these findings. Early clinical trials with rolipram demonstrated encouraging antidepressant effects in patients with major depressive disorder, while low dose roflumilast, a second-generation PDE4 inhibitor, has been shown to improve verbal and working memory in individuals with schizophrenia and healthy older adults (Zeller et al., 1984; Gilleen et al., 2021; Blokland et al., 2019b). In more recent trials, treatment with ibudilast, a non-selective PDE inhibitor with PDE4 activity, was shown to significantly reduce brain atrophy in a Phase II clinical trial in patients with progressive MS (Fox et al., 2018). Collectively, these findings support the therapeutic potential of PDE4 inhibitors across a range of neurological and psychiatric conditions.

Several PDE4 inhibitors, such as roflumilast (Daliresp), apremilast (Otezla), and crisaborole (Eucrisa), have been successfully developed and approved for peripheral inflammatory conditions, demonstrating that PDE4 is a clinically validated and druggable target (Li et al., 2018). However, despite extensive efforts to develop PDE4 inhibitors for CNS disorders, clinical success has been hampered by various issues, including poor brain penetration, suboptimal pharmacokinetics, and dose-limiting side effects, especially nausea and diarrhea (McDonough et al., 2020; Robic et al., 1999; Kang et al., 2023; Krause and Kühne, 1988; Sanftner et al., 2009). To address these limitations, several strategies have been explored, including the development of subtype-selective inhibitors and compounds with refined binding properties (Burgin et al., 2010; Naganuma et al., 2009; Barnette et al., 1995). While subtype-selective compounds have demonstrated improved tolerability, effective modulation of disease-relevant signaling pathways may require broader inhibition across subtypes, which subtype-selective agents may not achieve. To date, no broad-spectrum PDE4 inhibitor has shown clinical efficacy in CNS disorders without being limited by intolerable side effects or insufficient brain penetration.These challenges highlight the need for next-generation PDE4 inhibitors with improved brain penetration and tolerability.

In this report, we describe the *in vitro* and *in vivo* pharmacological and safety profile of 11h, a potent, selective, and brain-penetrant PDE4 inhibitor that demonstrates robust anti-neuroinflammatory activity and improved tolerability in preclinical models. Compound 11h was rationally designed to address the limitations of first- and second-generation PDE4 inhibitors by optimizing molecular structure and pharmacokinetic properties to enhance CNS exposure and achieve therapeutic efficacy at lower doses (Vadukoot et al., 2020). These modifications aim to expand the therapeutic window and reduce dose-limiting adverse effects commonly associated with PDE4 inhibition. Molecular docking studies demonstrated that 11h binds with high affinity to a pocket within the catalytic domain of PDE4, characterized by strong hydrophobic and electrostatic complementarity, a finding that may contribute to its optimized binding profile. The data presented herein characterize the pharmacokinetic properties and potent anti-inflammatory activity of 11h in rodent models, and demonstrate that the compound is safe and well-tolerated with repeat dosing. Notably, 11h exhibits no significant off-target activity, appears to lack cytotoxic or genotoxic potential, and does not elicit emesis even at supratherapeutic doses in rodent models. These findings highlight the favorable safety and tolerability profile of 11h and its potential as a novel PDE4 inhibitor for CNS diseases.

## Materials and methods

### Schrödinger docking studies and protein reliability assessments

The three-dimensional crystal structure of human PDE4A10, a long isoform which consists of all known regulatory binding domains in the enzyme family, was obtained from the Protein Data Bank (PDB ID: 2QYK). Structural preparation was carried out using the Schrödinger Protein Preparation Wizard. This process involved modeling missing side chains and loops, optimizing the hydrogen bonding network, assigning protonation states consistent with physiological pH (∼7.4), and energy minimizing the structure to resolve steric clashes and ensure stability. To identify potential ligand binding sites, the protein was analyzed using Schrödinger’s SiteMap tool. Binding pockets were evaluated for druggability based on volume, enclosure, hydrophobic/hydrophilic balance, and hydrogen bonding potential. Five sites were identified, with Site 1 emerging as the most druggable (SiteScore = 1.045; volume = 433.9 Å^3^), and Sites 2–5 demonstrating progressively lower SiteScores and volumes.

11h was prepared for docking using LigPrep (Schrödinger Suite), which generated low-energy three-dimensional conformers and assigned appropriate protonation and ionization states at pH 7.0 ± 0.5. The molecule was found to contain three rotatable bonds. Molecular docking simulations were performed using the Glide module in standard precision (SP) mode. 11h was docked independently into each of the five identified binding sites using a rigid receptor and flexible ligand protocol. Docking poses were evaluated based on multiple criteria including GlideScore, Glide emodel, lipophilic and Coulombic interaction energies, hydrogen bonding contributions, and ligand efficiency metrics (size-normalized binding energy).

To ensure structural reliability of the PDE4 model, a comprehensive quality assessment was conducted. This included evaluation of Ramachandran outliers, identification of buried unsatisfied polar groups, and detection of steric clashes. Minor issues such as a steric clash involving GLN454 and several disallowed backbone dihedrals were addressed through targeted refinement, resulting in a robust model suitable for high confidence docking studies.

### Animal models

All animal procedures were approved by the Institutional Animal Care and Use Committee (IACUC) and relevant institutional ethical review boards at K2bio (Houston, TX), Temple University (Philadelphia, PA), Porsolt (France) and TheraIndx Life Sciences (India), and conducted in accordance with Animal Research: Reporting of *In Vivo* Experiments (ARRIVE) guidelines (Percie du Sert et al., 2020). Group sizes were determined by *a priori* power analysis (85% power) to ensure sufficient statistical rigor while minimizing animal use. Adult male C57BL/6 mice (6–8 weeks old; The Jackson Laboratory), Sprague-Dawley (SD) rats (6–8 weeks old; Charles River Laboratories), and male ferrets (1.09–1.87 kg; Marshall BioResources, USA) were used in the studies. Animals were acclimated to the housing environment for at least 1 week prior to experimentation. Mice were housed in groups of three to five in ventilated microisolator cages, and rats in groups of two to three per cage in individually ventilated cages, under a 12 h light/dark cycle (lights on at 07:00), with controlled temperature (22 °C ± 2 °C) and humidity (50% ± 10%). Both species had access to food and water *ad libitum*, with corncob bedding, nesting material, and enrichment. Ferrets were housed in pairs in stainless steel cages (20 °C–22 °C, 12 h light/dark cycle), fed commercial ferret chow and provided daily environmental enrichment. In studies where euthanasia was required, animals were euthanized humanely by intraperitoneal (IP) injection of an overdose of sodium pentobarbital (>150 mg/kg), followed by transcardial perfusion with ice-cold phosphate-buffered saline (PBS) to ensure death prior to tissue collection. All efforts were made to minimize suffering and distress throughout the study.

### Chemicals and reagents

11h was synthesized and initially characterized by the University of Texas at San Antonio, with process optimization and scale-up performed by LAXAI Life Sciences (India). High-performance liquid chromatography (HPLC) confirmed purity exceeding 97%. For *in vitro* studies, 11h was dissolved in 100% dimethyl sulfoxide (DMSO). For *in vivo* use, it was formulated in a vehicle consisting of 15% ethanol, 5% DMSO, 30% PEG-400, 10% Cremophor, and 40% saline. The same vehicle was used as control in corresponding studies. Additional reagents are described in each section below.

### cAMP inhibition assay

PDE4 inhibition by 11h was assessed using a luminescence-based cAMP assay (Pharmaron, Hong Kong, CN). Compound stock solutions were prepared in DMSO and stored at room temperature (RT) in a desiccator for up to 3 months or at −20 °C for long-term storage. Forty nanoliters of diluted compound were transferred into 384-well plates using an Echo acoustic liquid handler. Plates were centrifuged (1,000 rpm, 1 min), and 2 μL of 2× recombinant PDE4 enzyme in assay buffer was added. After 10 min equilibration at RT, the reaction was initiated with 2 μL of 2× cAMP substrate and incubated for 60 min. AMP-Glo™ Reagent I (4 μL) was added and incubated for 60 min, followed by 8 μL of AMP Detection Solution. Luminescence was recorded using an EnVision plate reader (PerkinElmer). Assays were performed in duplicate. Percent inhibition was calculated using:

$$\%\text{Inhibition}=100\;–\;100\times \left[\left(\text{Readout}-\text{LCave}\right)/\left(\text{HCave}-\text{LCave}\right)\right]$$

Dose-response curves were fitted using a four-parameter logistic model in XLfit (IDBS), and IC_50_ values were derived accordingly.

### *In vitro* inflammatory assay (RAW and IMG cells)

RAW 264.7 cells (Taciak et al., 2018) were obtained from ATCC, Manassas, VA, USA, immortalized adherent mouse microglial cells “IMG cells” (Banerjee et al., 2021) were obtained from Sigma-Aldrich; St. Louis, MO, USA; Cat # SCC-134, RRID:CVCL HC49. RAW 264.7 cells were grown in Dulbecco’s modified Eagle’s (DMEM), high glucose, GlutaMAXTM media supplemented with 10% FBS (ThermoFisher Scientific, Asheville, NC, USA, Cat # 10569044). IMG cells were cultured in High Glucose DMEM (Sigma Cat. No. D6546) with 10% heat-inactivated fetal bovine serum (Gibco™ Cat # 10082147), 1× L-Glutamine (Sigma Cat # TMS-002-C) and 100 U/mL penicillin/streptomycin (Gibco™ Cat # 15140148). RAW 264.7 cells (2.5 × 10^5^) and IMG cells (4 × 10^4^) were seeded in 24 well plates and allowed to equilibrate overnight at 37 °C and 5% CO_2_. The following day the seeding media was replaced with fresh growth media and the cells pre-treated 11h 0.1–10 μM, *n* = 3/4, (or drug vehicle, DMSO Sigma, St Louis, MO, USA, Cat # D2650). One hour after the addition of drug, the cells were challenged with LPS (10 ng/mL, *E. coli* O55:B5, Sigma, St Louis, MO, USA). Twenty-four hours later, culture media was collected to quantify markers of inflammation (TNF-α protein and nitrite). Cell viability was assessed by using the CellTiter 96® Aqueous One Solution Cell Proliferation Assay kit (Promega, Madison, WI, USA, Cat # G3580), cell viability readouts were generated on an Infinite® 200 Pro plate reader (Tecan). Secreted TNF-α protein levels were assessed by use of a selective ELISA (ELISA MAX™ Deluxe Set Mouse TNF-α, BioLegend, CA, USA, Cat # 430915), Optical Densities (ODs) were generated on a SpectraMAX PLUS 385 plate reader, sample ODs were compared to those of a standard curve for both the cytokine and nitrite concentrations, the analyte levels were determined by use of the software Softmax Pro 7.1 Nitrite levels were assessed by the use of the Griess Reagent System (Promega, Madison, WI, USA, Cat # G2930) or the Nitrate/Nitrite Fluorometric Assay Kit, (Abnova, Cat # KA1344). Statistical analysis was performed with GraphPad Prism v10.5.0. Assessments of the data for statistical outliers were made using the Rout test, data normality by use of the Shapiro-Wilk test, if data were determined not to be normally distributed non-parametric analysis were performed, if data were normally distributed ordinary one-way ANOVAs were performed. Data are presented as mean ± SEM, of n observations, statistical significance was defined as *p* < 0.05.

### Human PBMC stimulation and ELISA

Human peripheral blood mononuclear cells (PBMCs) were purchased from STEMCELL Technologies and isolated *via* density gradient centrifugation and resuspended at 3 × 10^6^ cells/mL in complete RPMI 1640 medium (10% fetal bovine serum (FBS), 2 mM L-glutamine). Cells were distributed into T25 or T75 flasks and treated with LPS (2 μL/mL; *E. coli* O111:B4) or vehicle, with or without 11h (10 nM-30 µM). After 24 h incubation at 37 °C with 5% CO_2_, supernatants were harvested and stored at −80 °C. TNFα levels were quantified using a human TNFα ELISA kit (0–1,000 pg/mL standard curve), and absorbance was measured at 450 nm. Cytokine concentrations were interpolated from the standard curve.

### LPS-induced neuroinflammation model and immunophenotyping

Adult male C57BL/6 mice (8–10 weeks old) received two subacute IP injections of LPS (1 mg/kg, 6 h apart) (Sumbria et al., 2016). Two days later, mice were treated with 11h (1 mg/kg, IP) or vehicle. After 48 h, mice were euthanized, and brains were processed into single-cell suspensions. Samples were centrifuged (10,000 × g, 10 min, 4 °C). Supernatants were stored at −80 °C for nitrite and PGE_2_ assays; ∼2 million cells were used for flow cytometry, with the remainder stored for quantitative polymerase chain reaction (qPCR). Cells were stained in PBS with 2% FBS using fluorophore-conjugated antibodies (Abcam) targeting TLR4, CD86 (M1 macrophages), CD206 (M2 macrophages), TNFα, IFNγ, IL-6, CD4, and CD8. Fluorescence-minus-one (FMO) and isotype controls were used. Data were acquired on a BD LSRFortessa™ and analyzed with FlowJo. Nitrite levels were measured using a Griess assay kit (Thermo Fisher), and PGE_2_ *via* ELISA (RayBiotech). For gene expression, RNA was extracted using RNeasy kits (Qiagen), converted to cDNA (Thermo Fisher), and analyzed *via* SYBR Green-based qPCR (QuantStudio™). mRNA included *CXCL10*, *TNFα*, *NFΚβ*, *MYD88*, *IL1R1*, *FBL*, and *TRAAD*, normalized to *GAPDH*. Flow cytometry data were analyzed by one-way ANOVA with Bonferroni *post hoc* tests. mRNA expression comparisons (11h vs. LPS-only) used unpaired two-tailed t-tests with Welch’s correction. *p* < 0.05 was considered significant.

### CYP450 inhibition assay

11h was assessed for inhibition of CYP1A2, CYP2A6, CYP2C9, CYP2C19, CYP2D6, and CYP3A4 using recombinant human isoforms (Eurofins Panlabs, Missouri, USA). Assays were performed in duplicate at 0.1 and 10 μM, with IC_50_ determinations spanning 0.03–100 µM. Reactions were performed in phosphate buffer (pH 7.4) with an NADPH-generating system and <0.4 mg/mL bovine serum albumin. Fluorescence was measured at t = 0 and t = final. Percent inhibition was calculated relative to vehicle control. Non-linear regression (Hill equation) was used to determine IC_50_ values. Reference inhibitors were included to validate assay performance.

### Cardiac ion channel activity (QPatch assay)

Automated whole-cell patch-clamp assays were conducted using the QPatch platform (Sophion Bioscience) as part of the Comprehensive *in Vitro* Proarrhythmia Assay (CiPA) panel (Eurofins Panlabs). 11h (0.1–10 µM) was profiled for its ability to block three key human cardiac ion channels expressed in stable cell lines: Nav1.5 (sodium, HEK293), Cav1.2 (L-type calcium, HEK293), and hERG (potassium, CHO).

Nav1.5 Assay (CYL8004QP2DR): Cells were held under a voltage-clamp protocol consisting of a hyperpolarizing pulse to −120 mV (200 ms), followed by a depolarizing step to −15 mV (40 ms), a step to +40 mV (200 ms), and a ramp back to −80 mV (100 ms at 1.2 V/s), repeated every 5 s. Peak sodium current was measured at −15 mV. Data were filtered for seal quality, seal drop, and current amplitude. Percent inhibition was calculated by comparing mean current amplitude in the presence of 11h to control values collected 15 s before compound addition.

Cav1.2 Assay (CYL8051QP2DR): From a holding potential of −90 mV, inward calcium current was evoked by a 100 ms pulse to −60 mV followed by a 50 ms step to +10 mV. This sequence was repeated three times every 20 s for each test concentration. Current amplitude was monitored over time, and percent block was calculated by comparing the mean current amplitude in the presence of 11h to that of the pre-treatment control.

hERG Assay (CYL8038QP2DR): Cells were held at −80 mV and a 500 ms step to −40 mV was used to measure leak current, which was subtracted in real time. Cells were then depolarized to +40 mV for 500 ms and returned to −80 mV using a 100 ms ramp to elicit hERG tail currents. This protocol was repeated every 8 s. The maximum tail current amplitude was measured, and the degree of block was determined by comparing current amplitude before and after compound application.

In all assays, up to three concentrations of 11h were applied sequentially to each cell, and data were collected in duplicate. The percent inhibition of current was calculated by normalizing test compound amplitudes to pre-treatment control values. Assay quality was ensured through standard internal filtering criteria and reference compound validation.

### Mutagenicity and bacterial cytotoxicity (AMES test)

The mutagenic potential of 11h was evaluated using Ames assays (Eurofins Panlabs) with tester strains TA98, TA100, TA1535, and TA1537, with and without S9 metabolic activation. Cytotoxicity was assessed by bacterial growth inhibition at 0.6–100 μM. OD_650_ < 60% of control was considered cytotoxic. For mutagenicity, cultures were incubated with 11h and OD_430_/OD_570_ > 1 was recorded as a positive signal. Statistical significance was determined using Fisher’s exact test (one-tailed). Mitomycin C and other standard mutagens were used as positive controls.

### *In vitro* binding and enzyme profiling assays

11h was screened in a panel of binding and enzyme assays to predict potential adverse effects or alternative mechanism of action (Eurofins Cerep, FR). The compound was tested in duplicate wells at concentrations of 100 nM and 10 µM. Cell membrane homogenates were incubated with the appropriate radioligand in the absence or presence of the 11h in buffer. Nonspecific binding was determined in the presence of a specific agonist or antagonist for the target. Following incubation, the samples were filtered rapidly under vacuum through glass fiber filters presoaked in a buffer and rinsed several times with an ice-cold buffer using a 48-sample or 96-sample cell harvester. The filters were counted for radioactivity in a scintillation counter using a scintillation cocktail. Compound binding was calculated as a % inhibition of the binding of a ligand specific for each target. Compound enzyme inhibition effect was calculated as a % inhibition of control enzyme activity.

### Pharmacokinetic and tissue distribution studies

Pharmacokinetic profiling of 11h was performed using a single-dose cassette study design in rats (TheraIndx Lifesciences, India). Male SD rats (6 weeks old, *n* = 3 per group) received 11h *via* either intravenous (IV, 1 mg/kg) or oral (1 or 10 mg/kg) administration. Blood samples were collected at eight time points (0.083, 0.25, 0.5, 1, 2, 4, 8, and 24-h post-dose) under fasted conditions. Plasma was separated by centrifugation and stored at −80 °C until analysis. Plasma concentrations of 11h were quantified using a fit-for-purpose LC-MS/MS method. Pharmacokinetic parameters, including maximum plasma concentration (C_max_), area under the curve from time zero to infinity (AUC_0_), and time to reach maximum concentration (T_max_), were calculated using non-compartmental analysis.

A 5-day oral repeat-dose study was conducted to evaluate the multi-day pharmacokinetics of 11h. Male SD rats (6 weeks old, n = 6) received once daily oral gavage of 11h (10 mg/kg). Blood was collected on Day 1 at 30 min, 1, 2, 4, 6, and 8 h post-dose. On Day 5, samples were collected at 5, 15, 30 min, 1, 2, 4, 8, and 24 h post-dose. Blood samples (∼100 µL) were drawn *via* retro-orbital or saphenous vein collection into tubes containing 10% K_2_EDTA, gently mixed, placed on ice, and centrifuged at 10,000 rpm for 10 min. Plasma was harvested and stored at −80 °C. Concentrations of 11h were measured by LC-MS/MS. Noncompartmental pharmacokinetic analysis was performed using Phoenix WinNonlin to determine C_max_, T_max_, and AUC_0_.

A separate tissue distribution study was conducted to evaluate the biodistribution of 11h following a single oral dose. Male SD rats (6 weeks old, *n* = 6) received 11h by oral gavage, and were euthanized at three key time points representing the absorption phase (pre-C_max_), peak plasma concentration (C_max_), and elimination phase. At each time point, blood and tissues, including brain, heart, lung, liver, kidney, stomach, small intestine, large intestine, spleen, and pancreas, were collected. Tissue samples were processed and analyzed using a validated LC-MS/MS method. Plasma and tissue concentrations were reported for each time point, and tissue-to-plasma concentration ratios were calculated to assess drug distribution. Descriptive statistics (mean ± standard deviation) were used to summarize plasma and tissue concentration data.

Comparisons between groups or time points were not statistically tested in these studies, as the primary objective was to determine pharmacokinetic parameters rather than assess treatment effects. For all LC-MS/MS-based bioanalytical data, sample acceptance criteria were based on standard bioanalytical method validation guidelines (e.g., within ±15% of nominal values for accuracy and precision, except at the lower limit of quantification, where ±20% was acceptable).

### Red blood cell partitioning assay

11h was incubated in fresh whole blood, followed by centrifugation to separate plasma. Plasma and whole blood concentrations were determined using a validated LC-MS/MS method. Chromatographic separation was carried out on a Shimadzu LC-40D XS system coupled to a SCIEX Triple Quad 4500 mass spectrometer (Pharmaron, CN). Samples were injected onto an XSelect HSS T3 column (2.5 µm, 2.1 × 50 mm) maintained at 40 °C under standard conditions. Data were analyzed by calculating the blood-to-plasma partition ratio (B/P) for 11h, defined as the concentration in whole blood divided by the concentration in plasma. Ratios were computed using mean values from replicate measurements. These values provide an indication of the compound’s distribution between cellular and plasma compartments and support pharmacokinetic interpretation of its *in vivo* behavior.

### Maximum tolerated dose and repeat-dose tolerability studies

A maximum tolerated dose (MTD) study was conducted in adult male C57BL/6 mice (8 weeks old). Mice were acclimated for 72 h under standard laboratory conditions with access to food and water *ad libitum*. General health was monitored during the acclimation period, and baseline body weights and behavioral assessments were recorded prior to dosing. Animals received a single oral dose of 11h by gavage, administered in descending order from the highest to the lowest dose group. Mice were observed continuously for 30 min following dosing, then monitored daily for 7 days. Clinical observations included behavioral changes, grooming abnormalities, aggression, lesions, body condition scoring, food intake, ≥10% weight loss, unresolved health issues, and mortality.

In a repeat-dosing study, adult male SD rats (*n* = 4) were acclimated under the same conditions as described above. Following baseline assessments, rats received once daily IP injections of 11h (1.25 mg/kg) for 28 consecutive days. Animals were observed for 30 min post-injection and monitored daily for up to 10 weeks from the initial dose using the same clinical criteria described above.

In both studies, tolerability was defined as the resolution of any transient adverse effects within 24 h of symptom onset. No preemptive euthanasia or medical intervention was provided unless animals met predefined humane endpoints.

### Assessment of emesis and TNFα expression in ferrets

An emesis induction study was conducted in ten naïve male ferrets (*Mustela putorius furo*) to evaluate the emetic potential of 11h (Porsolt, France). The emesis assay was performed using a validated protocol adapted from Gardner et al. (1995). Sixty minutes prior to compound administration, ferrets were placed in individual stainless-steel cages (40 × 50 × 34 cm) with grid floors for habituation. Ferrets were dosed orally with 11h at 0.5, 1.8, 6.4, 12.8, or 19.2 mg/kg, or with ibudilast (10 mg/kg, Sigma-Aldrich), a non-selective PDE4 inhibitor known to induce gastrointestinal side effects. Each dose was tested in a separate group of five animals (*n* = 5/group), totaling six experimental groups. The study was conducted in three consecutive test sessions, each comprising two dose groups, with a minimum washout period of 1 week between sessions. Following dosing, animals were observed for a 2-h period for emetic responses. Parameters recorded included the number of ferrets exhibiting retching or vomiting, latency to first retch and first vomit, total number of retches and vomits, number of discrete emetic episodes, and presence of severe behavioral side effects. Retching was defined as rhythmic respiratory movements against a closed glottis, and vomiting as the forceful expulsion of upper gastrointestinal contents. In addition to emetic endpoints, stereotypical behaviors associated with nausea - including licking, backward walking, and clawing or scratching at the mouth - were recorded for each animal during 30-min intervals, including latency to first occurrence and number of animals affected.

At the conclusion of each 2-h evaluation period, blood samples were collected from all ferrets (*n* = 5 per group; six groups total) *via* cranial vena cava puncture using sterile disposable syringes. Approximately 0.5 mL of blood was drawn from each animal (totaling 30 samples) and transferred into pre-labeled tubes containing potassium (K_2_) EDTA as an anticoagulant. Tubes were sealed, gently agitated, and stored on ice until centrifugation, which occurred within 30 min of collection. Samples were centrifuged at 2000 *g* for 15 min at 4 °C. The resulting plasma was aliquoted into two polypropylene tubes (∼100 µL each) and stored at −20 °C until further analysis. Plasma samples were subsequently shipped to Eve Technologies Corporation (Alberta, Canada), where TNFα concentrations were quantified using a Nori Ferret TNFα ELISA kit (Genorise, USA). Cytokine levels were normalized to internal standards according to the manufacturer’s protocol.

### Elevated plus and zero maze behavioral assessment

Anxiety-like behavior was assessed in adult male rats (8 weeks old) using the Elevated Plus Maze (EPM) and Elevated Zero Maze (EZM) following repeated drug administration (Temple University). Rats received daily IP injections of 11h (0.375 mg/kg), rimonabant (3 mg/kg; Sigma-Aldrich), or vehicle for seven consecutive days. To minimize novelty-induced stress, animals were habituated to the behavioral testing room for 30 min on the day prior to testing. On Day 7, each rat was placed at the center of the EPM, facing an open arm, and allowed to explore freely for 5 min. The EPM consisted of two open and two closed arms elevated 50 cm above the floor. Immediately following EPM testing, rats were placed in the EZM, a circular elevated platform divided into four equal quadrants alternating between open and enclosed sections. Each rat was placed at the junction of an open and enclosed arm and allowed to explore the maze for 5 min. All sessions were conducted under low-light conditions and recorded using overhead video for blinded analysis. Behavioral measures, including time spent in open arms, number of open arm entries, and total distance traveled, were quantified using automated tracking software. Differences between groups were analyzed using a one-way ANOVA, followed by Bonferroni’s *post hoc* test to compare means across the three treatment groups. Statistical significance was defined as p < 0.05. Data are presented as mean ± SEM unless otherwise specified.

### Forced swim test (FST)

Adult male SD rats (*n* = 8 per group) received daily IP injections of 11h (0.375 mg/kg) for 21 consecutive days. On the final day of treatment, 11h was administered 15 h prior to the forced swim test (Temple University). All behavioral testing was conducted during the light phase of the cycle. On each test day, animals were acclimated to the behavioral testing room for at least 1 h prior to the start of the assay. The FST was conducted using transparent cylindrical swim chambers (height: 45 cm, diameter: 20 cm) filled to a depth of 15–18 cm with water maintained at 27 °C ± 1 °C. On Day 1 (pre-test), rats were individually placed in the cylinder for 15 min. Following the pre-test, animals were gently dried and returned to their home cage. When group housing was used, pre-tested rats were housed separately to avoid social stress. Temporary holding cages with fresh bedding were used as needed to prevent mixing of pre- and post-test animals. Water temperature and depth were monitored and adjusted as necessary between each trial to ensure consistency. Twenty-four hours after the pre-test, animals underwent the test session. The swim cylinder was prepared as described above. Each rat was placed individually into the water, and behavior was video recorded for 5 min. Between subjects, water was replaced to maintain cleanliness and temperature control. Differences between groups were analyzed using an unpaired two-tailed t-test with Welch’s correction. Statistical significance was defined as *p* < 0.05. Data are presented as mean ± SEM unless otherwise specified.

## Results

### 11h exhibits potent, selective PDE4 inhibition

**FIGURE 1:**
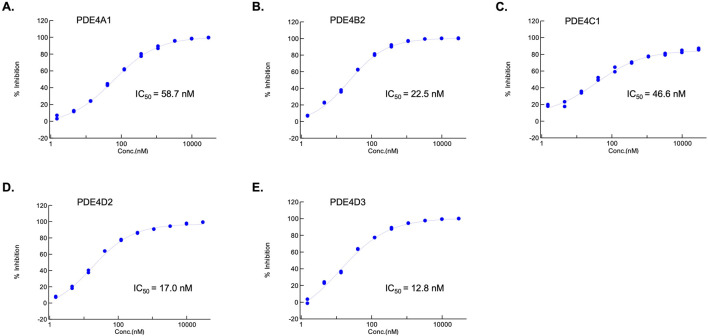
Potent, dose-dependent inhibition of multiple PDE4 isoforms by 11h. Isoform-specific activity of 11h was assessed in a high-throughput cAMP inhibition assay against PDE4A1, PDE4B2, PDE4C1, PDE4D2, and PDE4D3, to determine IC_50_ values. **(A–E)** Dose-response curves show complete inhibition of PDE4A1, PDE4B2, PDE4D2, and PDE4D3, and partial inhibition of PDE4C1. Data represent duplicate measurements normalized to assay controls and fitted with a four-parameter logistic regression model.

PDE4 inhibition by 11h was evaluated using a high-throughput, luminescence-based cAMP assay, enabling determination of isoform-specific IC_50_ values. 11h demonstrated potent, dose-dependent inhibition of multiple PDE4 isoforms in a high-throughput cAMP inhibition assay (Figures 1A–E). Full inhibition was observed for PDE4A1 (IC_50_ = 58.7 nM), PDE4B2 (IC_50_ = 22.5 nM), PDE4D2 (IC_50_ = 17.0 nM), and PDE4D3 (IC_50_ = 12.8 nM), while ∼80% inhibition was noted for PDE4C1 (IC_50_ = 46.6 nM). IC_50_ values were calculated from dose-response curves fitted with a four-parameter logistic model, indicating robust selectivity and potency across functionally relevant PDE4 subtypes.

### 11h exerts potent anti-inflammatory activity in mouse and human immune cells

**FIGURE 2:**
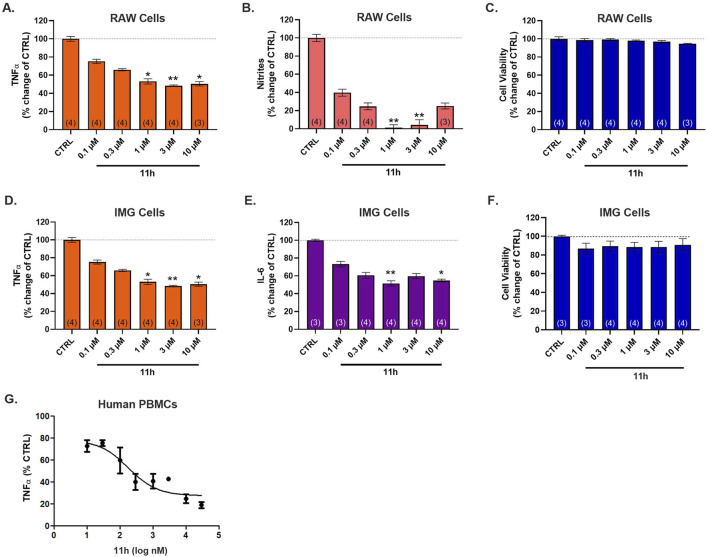
11h reduces LPS-induced pro-inflammatory cytokine production in mouse and human immune cells without affecting viability. RAW 264.7 (macrophage-like) and IMG (microglia-like) mouse cells were stimulated with LPS (10 ng/mL) and treated with increasing concentrations of 11h. In RAW cells, 11h significantly reduced **(A)** TNFα and **(B)** nitrite levels without impacting **(C)** cell viability. In IMG cells, 11h likewise reduced **(D)** TNFα and **(E)** IL-6, with no effect on **(F)** viability. **(G)** In human PBMCs, 11h decreased LPS-induced TNFα release. Data are presented as mean ± SEM (*n* = 4 biological replicates unless otherwise indicated). Significance: *p ≤ 0.05, **p ≤ 0.01 vs. control.

PDE4 inhibitors are well-recognized for their potent anti-inflammatory effects in immune cells such as macrophages and microglia, the latter playing a central role in maintaining brain homeostasis and mediating injury repair (Singh, 2022; Chen Y. et al., 2025). To evaluate the anti-inflammatory activity of 11h, mouse RAW 264.7 macrophages and IMG microglia-like cells were pretreated with vehicle (DMSO) or increasing concentrations of 11h (0.1–10 µM) 30 min before stimulation with 10 ng/mL LPS, and cytokine levels in the supernatants were measured 2 h later using ELISA or a fluorescence-based assay. Treatment with 11h markedly reduced the production of pro-inflammatory mediators, including TNFα, IL-6, and nitrites, in a dose-dependent manner without affecting cell viability (Figures 2A–F). In parallel, isolated human peripheral blood mononuclear cells (PBMCs) were treated with LPS (2 μL/mL) or vehicle (DMSO) in the presence or absence of 11h (10 nM-30 µM) for 24 h, and TNFα levels in the supernatants were measured by ELISA. 11h effectively suppressed LPS-induced TNFα release, further demonstrating its immunomodulatory activity across species (Figure 2G).

### 11h attenuates cellular and transcriptional markers of neuroinflammation in mice

**FIGURE 3:**
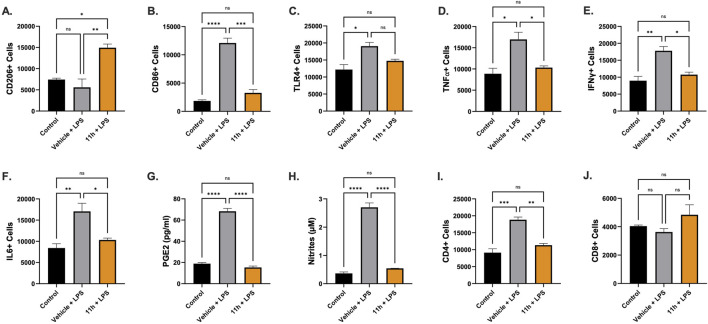
11h attenuates LPS-induced neuroinflammation in mice. C57/Bl6 mice received two subacute doses of LPS (1 mg/kg, IP) followed 2 days later by 11h (1 mg/kg, IP) or vehicle. Brain tissue was analyzed *via* flow cytometry 2 days after treatment. 11h increased anti-inflammatory M2 macrophages (CD206^+^; **(A)** and reduced pro-inflammatory M1 macrophages (CD86^+^; **(B)** Engagement of the LPS–TLR4 pathway was evidenced by elevated TLR4^+^ cell counts, which were modestly but not significantly reduced by 11h **(C)**. Expression of TNF-α **(D)**, IFN-γ **(E)**, IL-6 **(F)**, PGE_2_ **(G)**, and nitrites **(H)** was decreased following 11h administration. In addition, 11h reduced CD4^+^ T-cell numbers in the brain **(I)** without affecting CD8^+^ T-cell counts **(J)**. Data are presented as mean ± SEM (*n* = 6/group); *p < 0.05, **p < 0.01, ***p < 0.001, ****p < 0.0001.

The *in vivo* anti-inflammatory activity of 11h was assessed using a murine model of LPS-induced neuroinflammation. C57BL/6 mice were administered two subacute IP injections of LPS (1 mg/kg) to induce a robust central immune response (Sumbria et al., 2016). Three days post-challenge, animals received a single IP dose of 11h (1 mg/kg) or vehicle control. Brain tissue was collected 48 h after treatment and analyzed by flow cytometry. Compared to the vehicle-treated LPS group, 11h significantly increased the number of anti-inflammatory CD206^+^ (M1) macrophages (Figure 3A), while reducing pro-inflammatory CD86^+^ (M2) macrophages (Figure 3B), suggesting a shift in immune cell phenotypes. 11h treatment did not significantly reduce TLR4^+^ cell counts (Figure 3C), although a downward trend was observed. 11h significantly reduced the number of cells expressing TNFα, IFNγ, IL-6, PGE_2_, and nitrites (Figures 3D–H), and also decreased CD4^+^ T cell counts (Figure 3I), while having no effect on CD8^+^ T cells (Figure 3J).

**FIGURE 4:**
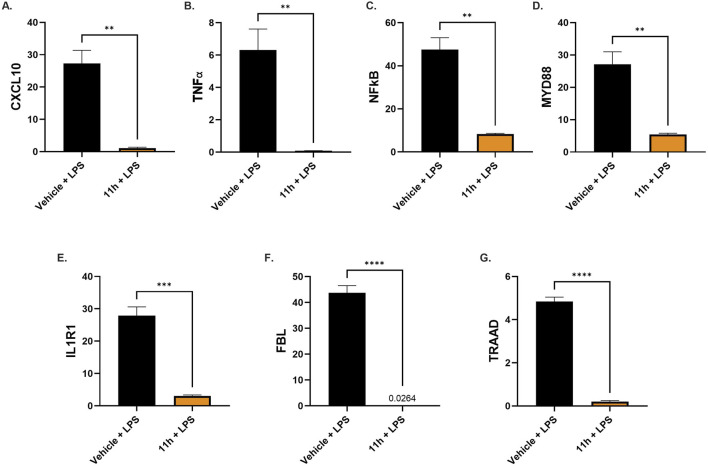
11h strongly downregulates pro-inflammatory gene expression in the MYD88 pathway (all *p* < 0.01). RT-qPCR analysis of brain samples from the same study as in Figure 3 showed that 11h significantly reduced the mRNA expression levels of key inflammatory genes involved in MyD88-dependent signaling, including **(A)** *CXCL10*, **(B)** *TNFα*, **(C)** *NFkB*, **(D)** *MYD88*, **(E)** *IL1R1*, **(F)** *FBL*, and **(G)** *TRAAD*. Data shown represent relative mRNA expression levels and are presented as mean ± SEM (*n* = 6/group). **p < 0.01, ***p < 0.001, ****p < 0.0001.

Stimulation of TLR4 receptors triggers downstream activation of myeloid differentiation primary response 88 (MyD88), an adaptor protein that recruits interleukin-1 receptor–associated kinases (IRAKs) and TNF receptor–associated factor 6, ultimately leading to activation of NF-κB and MAPK signaling pathways and driving the transcription of pro-inflammatory cytokines in microglia and other CNS-resident cells (Chen H. et al., 2025; Vetreno et al., 2021). Elevated intracellular cAMP levels have been shown to inhibit MyD88-dependent signaling, reducing IRAK phosphorylation and NF-κB nuclear translocation, thereby suppressing cytokine production (Ernst et al., 2019). Given that PDE4 inhibition by 11h elevates cAMP, we hypothesized that its anti-inflammatory effects may, at least in part, result from modulation of the MyD88 signaling cascade. To test this, RT-qPCR analysis was performed on mouse brain tissue collected from the same LPS study described above. Quantitative analysis of relative mRNA expression levels revealed significant downregulation of the entire MyD88 signaling axis - from upstream receptors (*IL1R1*) to core adaptors (*MYD88*, *TRAAD*), downstream effectors (*NFκB*), and inflammatory outputs (*TNFα*, *CXCL10*) (Figure 4).

Together, these findings demonstrate that 11h mitigates inflammation by suppressing pro-inflammatory immune cell activation and modulating key transcriptional regulators within pro-inflammatory signaling pathways.

### Comprehensive in vitro safety screening reveals low off-target liability for 11h

**FIGURE 5:**
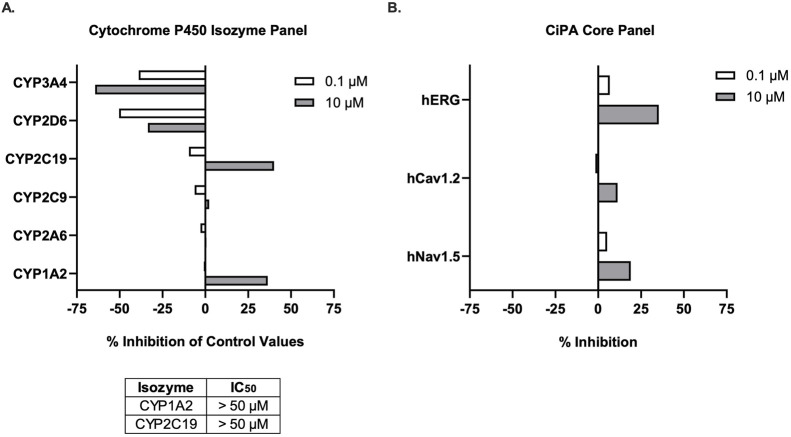
*In vitro* safety pharmacology profile of 11h. **(A)** 11h showed no meaningful inhibition of six major human CYP450 isoenzymes at 100 nM. Moderate inhibition of CYP1A2 and CYP2C19 was observed at 10 μM, but IC_50_ values for both exceeded 50 μM, indicating a low potential for clinically relevant CYP-mediated drug-drug interactions. **(B)** 11h did not inhibit hERG, Cav1.2, or Nav1.5 ion channels at either 10 nM or 10 μM, suggesting minimal proarrhythmic liability.

The safety pharmacology and off-target profile of 11h were assessed using a comprehensive panel of *in vitro* assays (Crespi et al., 1997). Given the critical importance of metabolic stability and drug-drug interaction potential for CNS therapeutics intended for chronic use, we first evaluated cytochrome P450 inhibition across six major human isoforms: CYP1A2, CYP2A6, CYP2C9, CYP2C19, CYP2D6, and CYP3A4. At 100 nM, 11h did not significantly inhibit any of the tested isoenzymes (Figure 5A). At 10 μM, moderate inhibition was observed for CYP1A2 (34.5%) and CYP2C19 (40.1%), while all other isoforms remained unaffected. Subsequent IC_50_ determinations for CYP1A2 and CYP2C19 yielded values > 50 μM, indicating a low risk of clinically relevant CYP-mediated drug-drug interactions at anticipated therapeutic concentrations.

Cardiac safety was evaluated using the Comprehensive *In Vitro* Proarrhythmia Assay (CiPA), which assessed the activity of 11h against three key human cardiac ion channels: hERG, Cav1.2, and Nav1.5. Given that PDE4 inhibitors have historically shown cardiovascular effects (Holbrook and Coker, 1991) and many neuropsychiatric drugs carry cardiac warnings, this evaluation was essential to demonstrate 11h′s therapeutic advantage. Importantly, 11h showed no significant inhibition of any channel at either 10 nM or 10 µM (Figure 5B), suggesting a minimal risk for QT interval prolongation or drug-induced arrhythmias.

The genotoxic potential of 11h was rigorously assessed using the bacterial reverse mutation (AMES) assay, a regulatory requirement for preclinical safety assessment. This assessment was critical because (1) PDE4 inhibitors are often chronically administered for neurological disorders, and (2) positive mutagenicity would preclude clinical development. We tested four standard *Salmonella typhimurium* strains (TA98, TA100, TA1535, and TA1537) with/without metabolic activation (rat liver S9 fraction), covering frameshift, base-pair substitution, and oxidative damage detection. (Maron and Ames, 1983). 11h showed no concentration-dependent mutagenicity (1.25–100 µM) in any strain, with or without S9 activation (Supplementary Table 1). A very weak positive response (p < 0.05) was detected in strain TA1537 with S9 activation; however, this signal was not accompanied by cytotoxicity or corroborated by results from other strains. No cytotoxicity was observed at any concentration tested, supporting the conclusion that 11h has low genotoxic risk under physiological conditions.

The off-target binding profile of 11h was evaluated using two complementary *in vitro* binding assays targeting neurologically relevant receptors and psychoactive drug targets. In a broad screen of over 100 CNS-associated proteins-including GPCRs, ion channels, transporters, and neurotransmitter receptors-11h showed only weak binding at high concentrations to the adenosine A_2_A (A2A) and progesterone (PR) receptors, with IC_50_ values > 10 µM (Supplementary Figure 1). No other significant off-target interactions were observed.

### 11h exhibits robust pharmacokinetics and strong brain exposure

**FIGURE 6:**
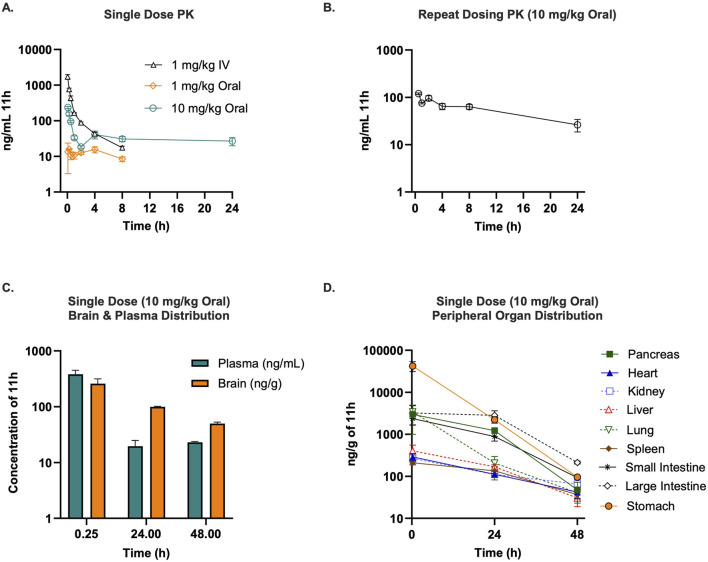
Pharmacokinetics and tissue distribution of 11h in rats after intravenous and oral administration. **(A)** Plasma concentration-time profiles of 11h following intravenous (1 mg/kg) or oral (1 or 10 mg/kg) administration (*n* = 3/group). After IV administration, 11h displayed high clearance (937.5 mL/min/kg), a short half-life (t_1_/_2_ = 2.6 h), and a large volume of distribution (V_d_ = 1904.6 L/kg). Oral dosing at 1 mg/kg produced moderate exposure (C_max_ = 18.7 ng/mL; AUC_inf = 182.2 ng h/mL; t_1_/_2_ = 7.3 h), whereas a 10 mg/kg oral dose produced higher systemic exposure (C_max_ = 236.3 ng/mL; AUC_inf = 916.8 ng h/mL; t_1/2_ = 313.6 h) with a prolonged terminal phase. **(B)** Repeat-dose pharmacokinetics after oral administration of 11h at 10 mg/kg once daily for 5 days (*n* = 6) showed approximately two-fold higher exposure on Day 5 (C_max_ = 158.2 ng/mL; AUC_inf = 2078 ng h/mL; t_1/2_ = 14.7 h), supporting once-daily dosing with drug accumulation. **(C)** Tissue distribution after a single 10 mg/kg oral dose (*n* = 3) demonstrated rapid and sustained brain penetration (C_max_ = 258.8 ng/g at 15 min) with brain levels remaining higher than plasma at 24 h and 48 h. **(D)** Peripheral tissue distribution revealed early high levels in gastrointestinal tissues, pancreas, and lungs, that declined by 48 h. Data are mean ± SEM.

The pharmacokinetic profile of 11h was characterized in rats using both IV (1 mg/kg) and oral (1 and 10 mg/kg) routes of administration to establish its absorption, distribution, and elimination properties, which are critical determinants for its development as a CNS therapeutic (Figure 6A; Table 1). Blood samples were collected at various time points to determine pharmacokinetic parameters, including clearance (CL), volume of distribution (Vd), maximum plasma concentration (C_max_), area under the curve (AUC), half-life (t_1/2_), and mean residence time (MRT). Following a 1 mg/kg IV dose, 11h exhibited high CL (937.5 mL/min/kg) and a short terminal half-life (t_1/2_ = 2.6 h). Furthermore, the large steady-state Vd of 1904.6 L/kg suggested extensive extravascular penetration. Oral administration at 1 mg/kg resulted in a C_max_ of 18.7 ng/mL and an AUC_inf_ of 182.2 ng h/mL, with an elimination t_1/2_ of 7.3 h. The CL and Vd were 91.9 mL/min/kg and 57.3 L/kg, respectively. Oral dosing at 10 mg/kg produced a C_max_ of 236.3 ng/mL at 4.8 min and an AUC_inf_ of 916.8 ng h/mL, with a secondary plasma peak at 4 h suggestive of enterohepatic recirculation, which may contribute to sustained drug exposure. The elimination t_1/2_ and MRT were 13.6 and 10.2 h, respectively. Drug levels were undetectable in blood by 48 h. Oral bioavailability was determined to be 11%.

**TABLE 1:** Single dose 11h pharmacokinetic parameters. (NRV*) indicates a value that was outside the instrument’s quantifiable range (i.e., below the lower or above the upper limit of detection). (ND) indicates a value which was not determined.

Parameter	IV (1 mg/kg)	PO (1 mg/kg)	PO (10 mg/kg)
T_1/2_ (h)	2.6	7.3	13.6
T_max_ (min)	1.8	11.4	4.8
C_0_ (ng/mL)	1918.1	ND	ND
C_max_ (ng/mL)	1714.7	18.7	236.3
AUC_0-t_ (ng⋅h/mL)	1,006.0	99.3	797.6
AUC_0_ (ng⋅h/mL)	1,073.7	182.2	916.8
MRT_last_ (h)	1.3	3.8	10.2
K_e_ (1/h)	0.3	0.1	0.1
CL (mL/min/kg)	937.5	91.9	6,681.4
V_d_ (L/kg)	3,604.4	57.3	NRV*
V_ss_ (L/kg)	1904.6	ND	ND

A separate cohort of rats received 10 mg/kg orally once daily for five consecutive days to assess repeat-dose pharmacokinetics (Figure 6B; Table 2). Oral administration of 11h at 10 mg/kg daily for 5 days demonstrated consistent pharmacokinetics with a steady increase in systemic exposure. On day 5, C_max_ reached 158.2 ng/mL at 15.6 min, and the AUC_inf_ was 2078 ng h/mL, approximately double the single-dose value. The elimination t_1/2_ was 14.7 h, with a blood CL of 91.25 mL/min/kg and a Vd of 92.6 L/kg, suggesting that once daily dosing with the drug is feasible.

**TABLE 2:** Five-day repeat dosing pharmacokinetic parameters for 11h. (ND) indicates a value which was not determined.

Pharmacokinetics (Day 5)		Ratio day 5: Day 1
T_1/2_ (h)	14.72	ND
T_max_ (min)	15.6	ND
C_max_ (ng/mL)	158.2	1.0
AUC_0-t_ (ng⋅h/mL)	1,334.6	1.9
AUC_0_ (ng⋅h/mL)	2078.1	ND
MRT_last_ (h)	8.32	2.1
K_e_ (1/h)	0.08	ND
CL (mL/min/kg)	91.25	ND
V_d_ (L/kg)	92.6	ND

Tissue distribution was evaluated in rats following a single 10 mg/kg oral dose, with drug levels quantified in brain, blood, and peripheral tissues at 15 min, 24 h, and 48 h post-dosing using a validated LC-MS/MS. After a single 10 mg/kg oral dose, 11h showed significant brain penetration, achieving a C_max_ of 258.8 ng/g at 15 min (Figure 6C; Table 3). Brain concentrations remained five-fold higher than blood plasma at 24 h and two-fold higher at 48 h, indicating sustained accumulation of drug in the brain. At 15 min post-dosing, moderate-to-high drug concentration levels were observed in the stomach, intestines, pancreas, and lungs, which levels diminished to insignificant levels by 48 h (Figure 6D; Table 3).

**TABLE 3:** Distribution of 11h (10 mg/kg PO) in brain, plasma, and peripheral tissues.

Time (h)	0.25	24	48
Concentration of 11h in tissue (ng/g) or plasma (ng/mL)			
Pancreas	2,983	1,227	47
Heart	291	111	42
Spleen	209	135	36
Stomach	42,301	2,222	96
Kidney	267	111	66
Liver	403	168	31
Lung	3,417	207	41
Large intestine	3,219	2,826	214
Small intestine	2,371	883	93
Brain	258.8	99.6	50.2
Plasma	381.4	19.6	23.0
B:P ratio	0.7	5.1	2.2

As drug accumulation in red blood cells (RBC) can influence distribution, clearance, and overall pharmacokinetics, the blood-to-plasma partition coefficient (K_b/p_) of 11h was evaluated using a standard blood cell partitioning assay. Whole blood samples from human, dog, rat, and mouse were used, and results were compared to chloroquine, a highly lipophilic compound known to accumulate in RBCs (Russo et al., 2024). Across all species tested, 11h exhibited consistently low K_b/p_ values relative to chloroquine, indicating minimal partitioning into RBCs (Supplementary Table 2).

### 11h is well-tolerated in rodents

**FIGURE 7:**
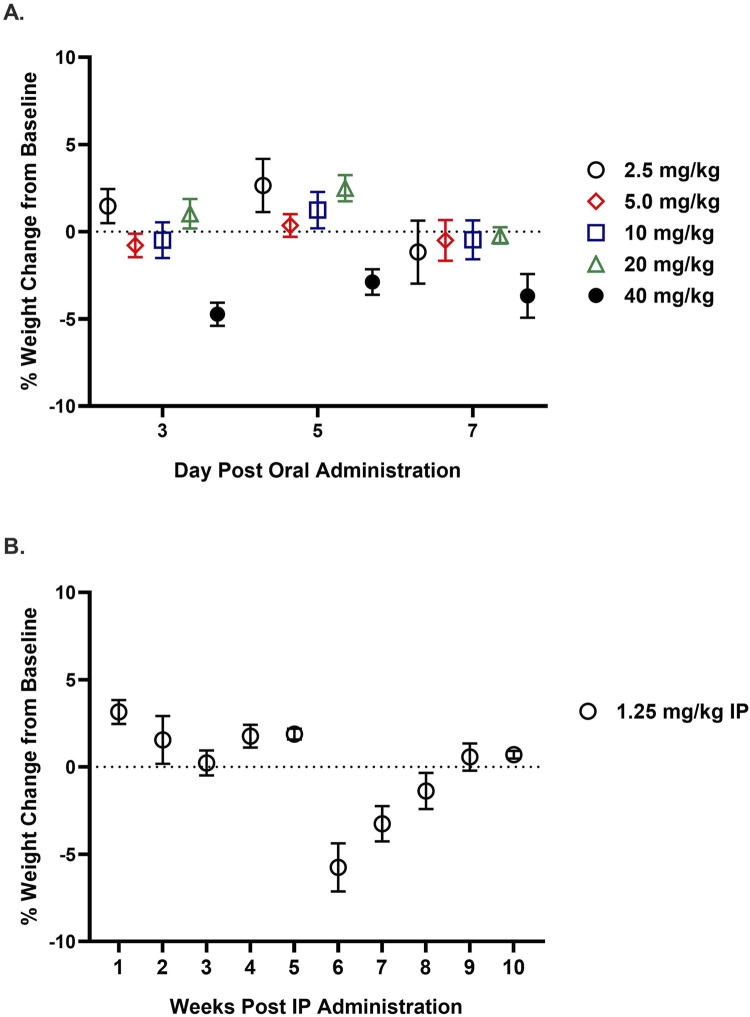
Effect of single and repeated dosing of 11h on rodent weight. **(A)** A maximum tolerated dose (MTD) study in mice after a single oral dose of 11h (2.5–40 mg/kg; *n* = 5/group). Mice were monitored for 7 days for behavior, body weight, food/water intake, and general health. No significant adverse effects were observed at any dose. A transient ∼5% weight loss at 40 mg/kg resolved by day 7 and did not meet the predefined toxicity threshold (≥10% loss), indicating 11h was well tolerated and an MTD was not reached. **(B)** Repeat-dose study, in rats receiving 11h (1.25 mg/kg IP, *n* = 5) once daily for 28 days. Body weight and general health were monitored for 10 weeks. Animals exhibited a transient ∼5% weight loss at week 6 that recovered by week 10, with no behavioral changes noted. Data are presented as mean ± SEM.

A maximum tolerated dose (MTD) study was conducted in mice using single escalating oral doses of 11h (2.5, 5, 10, 20, or 40 mg/kg) (Figure 7A). Animals were observed for behavioral and physiological signs of toxicity, including grooming, aggression, lesions, body conditioning, food and water intake, weight changes, and sudden death, with immediate monitoring for 30 min post-administration and daily assessments over a 7-day period. No significant signs of toxicity were observed at any dose, and animals remained healthy with normal behavior and eating habits. The highest dose (40 mg/kg) led to a transient body weight loss of approximately 5% by day 3, which did not meet the toxicity criterion of 10% weight loss. As no toxicity or intolerance was identified, the MTD of 11h could not be determined in this study.

In a separate study, the effects of repeat dosing with 11h on body weight were assessed in rats administered 1.25 mg/kg IP once daily for four consecutive weeks (Figure 7B). Animals were monitored for both behavioral changes and weight fluctuations over a 10-week period. A transient ∼5% reduction in body weight was observed at week 6, which fully resolved by week 10. No abnormal behaviors were noted throughout the study, suggesting the drug to be well-tolerated with repeat dosing.

### 11h reduces TNFα expression at non-emetic doses in ferrets

**FIGURE 8:**
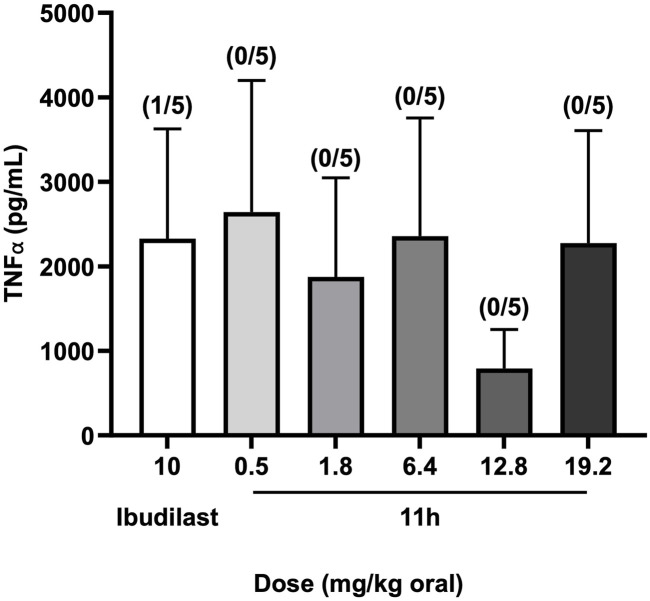
Emetic liability and anti-inflammatory activity of 11h in ferrets. Ferrets received single oral doses of 11h (0.5–19.2 mg/kg) or ibudilast (10 mg/kg) (*n* = 5 per group), and were monitored for signs of emesis (retching, salivation, vomiting), and other adverse effects. After the observation period, blood samples were collected and assessed for TNFα by ELISA. Bar graphs show TNFα levels for each treatment group with the number of animals that vomited indicated in parentheses above each bar. Ibudilast induced emesis in one of five animals, whereas 11h caused no vomiting at any tested dose. 11h also produced a non-linear, dose-dependent suppression of TNFα, with the 0.5 mg/kg dose achieving a reduction comparable to 10 mg/kg ibudilast. Data are presented as mean ± SEM.

To evaluate the emetic liability of 11h, a dose-ranging study was conducted in ferrets, the gold-standard model for drug-induced emesis owing to their robust vomiting reflex (Vetreno et al., 2021; Ernst et al., 2019). Animals received a single oral dose of 11h (0.5, 1.8, 6.4, 12.8, or 19.2 mg/kg) or the non-selective PDE4 inhibitor ibudilast (10 mg/kg), which is known to cause vomiting in humans (Fox et al., 2018). Ferrets were monitored for 2 h post-dosing for signs of emesis, including retching, vomiting, salivation, and other related behaviors. Notably, 11h did not induce vomiting at any dose tested (Figure 8, parentheses), although mild salivation was observed in animals receiving ≥1.8 mg/kg. In contrast, ibudilast triggered vomiting in one out of five animals within the first 5 min. These results suggest that 11h is better tolerated than both ibudilast and rolipram, with the latter reported to elicit emetic-like behaviors in rodents and vomiting in ferrets at doses as low as 0.1 and 3.0 mg/kg, respectively (McDonough et al., 2020; Crespi et al., 1997).

To evaluate the pharmacodynamic activity of 11h, ferret blood samples were collected at the end of the observation period and analyzed for expression levels of TNFα, a pro-inflammatory cytokine cpommonly used to assess pharmacodynamic activity for PDE4 inhibitors (Brideau et al., 1999). 11h reduced TNFα levels in a dose-dependent manner, with the lowest tested dose (0.5 mg/kg) exhibiting activity comparable to 10 mg/kg ibudilast (Figure 8, bar graphs). Although no statistically significant differences were detected between treatment groups (likely due to high within-group variability) these findings suggest that 11h exerts strong anti-inflammatory effects at well-tolerated, non-emetic doses.

### Chronic administration of 11h does not impair neurobehavioral responses in naïve rats

**FIGURE 9:**
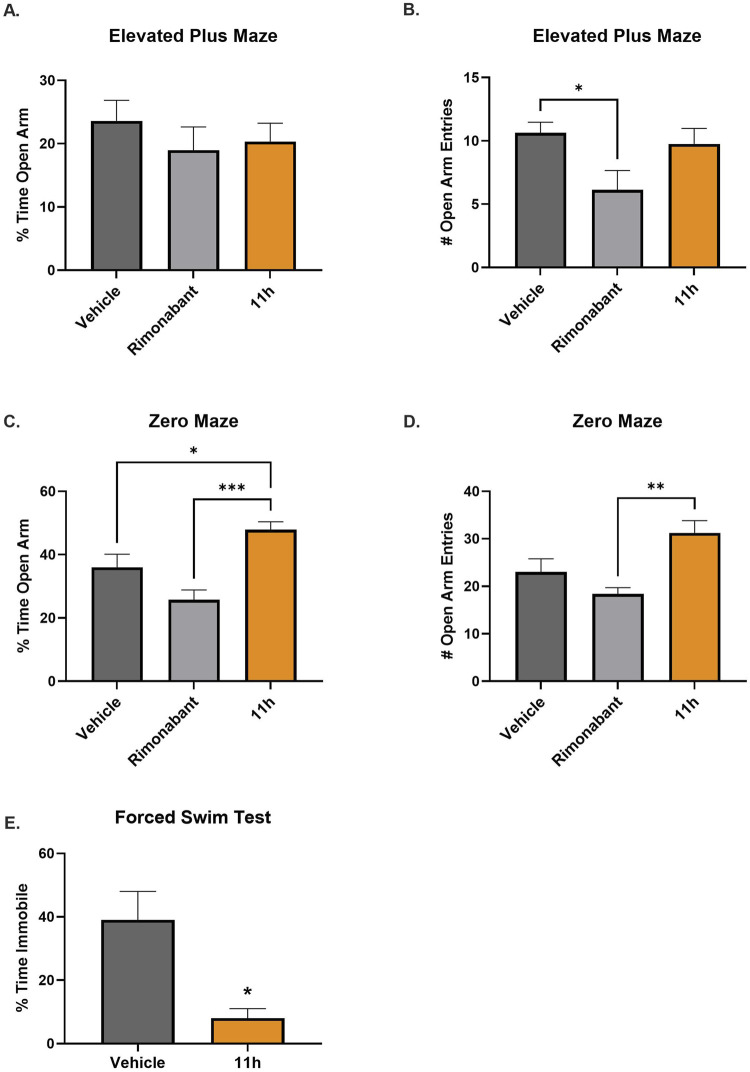
Neurobehavioral safety assessment in rats following repeat dosing with 11h. Rats received daily intraperitoneal injections of 11h (0.375 mg/kg), rimonabant (3 mg/kg), or vehicle for 7 days prior to testing in elevated plus and zero mazes. In the elevated plus maze, 11h produced no differences from vehicle in **(A)** time spent in or **(B)** entries into the open arms, whereas rimonabant significantly reduced open-arm entries. In the zero maze, 11h-treated animals showed significantly greater **(C)** time spent and **(D)** entries into the open arms compared to rimonabant-treated mice, with no difference from vehicle. **(E)** In a separate cohort, rats were treated with 11h (0.375 mg/kg, IP) or vehicle once daily for 21 days and evaluated in the forced swim test (FST). 11h treatment significantly decreased immobility duration. No lethargy, agitation, or abnormal behaviors were observed in any assay. Data are presented as mean ± SEM (*n* = 8/group); *p ≤ 0.05, **p ≤ 0.01, ***p ≤ 0.001.

To assess the neurobehavioral safety profile of 11h, we evaluated the effects of repeated dosing in rats using the elevated plus maze (EPM) and elevated zero maze (EZM), two well-validated assays for measuring anxiety-like behavior through open-arm avoidance (Walf and Frye, 2007; Braun et al., 2011). Rats were treated once daily for seven consecutive days with vehicle, 11h (0.375 mg/kg, IP), or rimonabant (3 mg/kg, IP), a cannabinoid 1 receptor (CB1R) inverse agonist associated with adverse psychiatric effects in humans (Moreira and Crippa, 2009). In the EPM, 11h-treated animals showed no significant differences compared to vehicle in time spent in the open arms (Figure 9A) or number of open-arm entries (Figure 9B). In contrast, rimonabant significantly reduced open-arm entries compared to vehicle, consistent with its known anxiogenic effects. In the EZM, 11h-treated rats spent significantly more time in the open arms compared to both vehicle- and rimonabant-treated animals (Figure 9C), and exhibited more open-arm entries than the rimonabant group (Figure 9D).

To further assess the neurobehavioral safety of 11h, a separate cohort of rats underwent the forced swim test (FST) following sub-chronic administration. The FST is a validated assay in which increased immobility is interpreted as a marker of behavioral despair (Slattery and Cryan, 2012). Rats were treated with 11h (0.375 mg/kg, IP) or vehicle once daily for 21 consecutive days, followed by FST assessment. Rats treated with 11h showed significantly reduced immobility compared to vehicle controls (Figure 9F), indicating an absence of depressant effects.

No signs of lethargy, agitation, or other abnormal behaviors were observed in 11h-treated rats across any of the three behavioral assays. Collectively, these results indicate that repeated dosing with 11h is well-tolerated and does not induce anxiety- or depressive-like behaviors in healthy rodents, supporting a favorable neuropsychiatric safety profile.

### Differential binding affinity and site preference of 11h across PDE4 pockets

**FIGURE 10:**
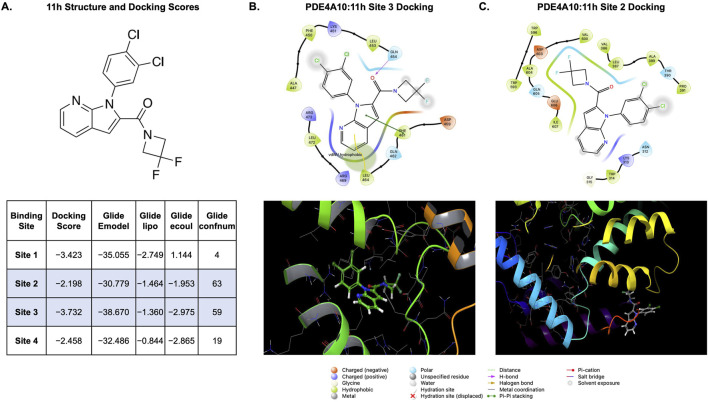
In silico interaction mapping of 11h across PDE4 binding sites. **(A)** Chemical structure of 11h (top) and comparative summary of docking metrics (bottom) across four predicted PDE4A10 binding sites. Metrics include Glide docking score, Emodel energy, lipophilic contribution (Lipo), electrostatic energy (Ecoul), and conformational sampling count (Confnum). Sites 2 and 3 emerged as the most favorable, with Site 2 offering higher conformational flexibility and Site 3 showing stronger hydrophobic and electrostatic interactions. **(B,C)** Top: Two-dimensional interaction diagrams depicting the key molecular interactions of 11h within Site 3 **(B)** and Site 2 **(C)**, highlighting van der Waals contacts (yellow line) and π–π stacking interactions (green line) in Site 3. Bottom: Corresponding three-dimensional docking views depicting ligand embedding and interaction networks. Site 3 shows deeper hydrophobic embedding and enhanced binding stability. All energy values are in kcal/mol. Glide confnum reflects the rank of sampled conformations; higher values suggest greater conformational flexibility.

Molecular docking analysis using Schrödinger Suite revealed that 11h exhibits variable binding affinities across the five identified PDE4 binding pockets, with notable differences in pose stability, interaction profiles, and ligand efficiency (Figure 10A). Among these, Site 3 demonstrated the most favorable binding characteristics, with a Glide docking score of −3.732 kcal/mol, the lowest (most negative) value observed across all tested sites. This was followed by Site 1 (−3.423 kcal/mol), Site 4 (−2.458 kcal/mol), and Site 2 (−2.198 kcal/mol), with Site 2 displaying the weakest predicted affinity despite greater conformational adaptability, as indicated by its higher Glide confnum (the conformation number of a ligand that has been docked into a receptor binding site using the GLIDE docking software). Quantitative docking metrics further supported Site 3 as the optimal binding location for 11h. This site exhibited the most favorable Glide emodel score (−38.670 kcal/mol), reflecting a stable receptor-ligand complex with minimized internal strain. The Glide lipophilic term (−1.360 kcal/mol) and Coulombic energy (−2.975 kcal/mol) at Site 3 suggested strong hydrophobic and electrostatic contributions to the binding interaction.

Structural analysis of the docked poses provided further insights into site-specific interactions. At Site 3, 11h was deeply embedded in a hydrophobic cleft, stabilized by π-π stacking interaction with phenylalanine (Phe461) residue, along with van der Waals contacts with leucine (Leu464) (Figure 10B). Electrostatic stabilization was moderate, reflected by the negative Glide ecoul value. Site 1, although moderately favorable, presented a shallower hydrophobic environment with fewer polar interactions. Minimal hydrogen bonding (Glide hbond = 0) and limited π-π stacking contributed to its weaker affinity compared to Site 3. In Site 2, the ligand adopted a deeper pose and formed more favorable electrostatic interactions with nearby Asp and Glu residues; however, this was not sufficient to compensate for weaker hydrophobic complementarity, resulting in a lower docking score (Figure 10C). Interestingly, this site supported greater ligand flexibility, as shown by the highest confnum (Rocque et al., 1997), indicating the ability of 11h to sample a broader conformational space. Site 4 showed the least favorable interaction profile. The ligand was positioned toward the edge of the pocket, with limited stabilizing contacts and reduced embedding. Both lipophilic and electrostatic scores were among the lowest, indicating a suboptimal fit and weak stabilization.

Assessment of ligand efficiency, defined as docking score normalized to ligand size, highlighted Site 3 as the most efficient binding environment for 11h (Glide Ligand Efficiency: -0.149). Site 2 produced the lowest efficiency (−0.088), reinforcing the conclusion that deeper electrostatic engagement alone does not ensure optimal binding.

## Discussion

In this study, we characterized the pharmacological profile, safety, and anti-inflammatory activity of 11h, a next-generation, brain-penetrant PDE4 inhibitor engineered to overcome the dose-limiting side effects that have hindered earlier compounds in this class. Our results demonstrate that 11h combines potent, broad-spectrum PDE4 inhibition with robust anti-inflammatory activity, favorable pharmacokinetics, low off-target liability, and a strong tolerability profile, most notably lacking the emetogenic and behavioral side effects that have constrained the clinical use of other PDE4 inhibitors.

Efforts to address the dose-limiting toxicities of this drug class have often focused on developing subtype-selective inhibitors, particularly those targeting PDE4B, which is highly expressed in the brain and implicated in neuroinflammation (Pearse and Hughes, 2016; Armstrong et al., 2024). Conversely, PDE4D is linked to emetic effects due to its expression in the area postrema in the brainstem (Mori et al., 2010). However, recent findings indicate that PDE4D inhibition may offer therapeutic benefits, such as pro-cognitive effects and enhanced remyelination, prompting interest in PDE4D-selective allosteric modulators with reduced emetic liability (Zhang et al., 2017; Paes et al., 2023; Miró et al., 2002). These advances challenge the assumption that PDE4D is solely responsible for nausea and suggest that overly selective approaches may sacrifice efficacy, given the complementary roles of PDE4B and PDE4D in neural and glial signaling. Thus, broad inhibition across multiple PDE4 isoforms may be necessary for addressing the complex pathophysiology of CNS disorders. One proposed mechanism underlying the emetic liability of PDE4 inhibitors involves high-affinity binding to the rolipram binding site, referred to as the high-affinity rolipram binding site (HARBS), located within the UCR2 domain of PDE4D. Binding to HARBS is thought to engage neural circuits in the brainstem that trigger nausea and vomiting (Duplantier et al., 1996; Boyd et al., 2021). As such, avoiding this site through structural modifications or selective engagement of non-HARBS regions may represent a promising strategy to preserve therapeutic activity while minimizing dose-limiting gastrointestinal side effects.

To better understand the binding mechanism of 11h to PDE4, we employed *in silico* molecular docking techniques to predict its binding site preferences and interaction profile. The long-form isoform PDE4A10 was selected as the structural model due to its inclusion of the conserved N-terminal regions UCR1 and UCR2, which are critical for dimerization and regulation of enzymatic activity. SiteMap analysis identified five putative binding pockets on PDE4A10, of which 11h demonstrated appreciable binding at four. Of these, Sites 2 and 3, which overlap with the catalytic domain of PDE4, were determined to have the most favorable binding kinetics, with Site 3 exhibiting the most stable and energetically preferred binding pose (Jacobitz et al., 1996; Souness and Rao, 1997). Docking at Site 3 was driven by extensive hydrophobic embedding, π-π stacking with aromatic residues, and moderate electrostatic stabilization. Although Site 2 supported greater conformational flexibility, it lacked sufficient hydrophobic contacts, leading to reduced binding efficiency. Importantly, neither Site 2 nor Site 3 significantly overlaps with the published HARBS for PDE4A and PDE4B (Rocque et al., 1997; Jacobitz et al., 1996), a distinction that may contribute to the improved tolerability profile of 11h and its reduced emetic liability (Burgin et al., 2010; Souness and Rao, 1997). These findings highlight a critical pharmacophore region within the catalytic domain that may drive isoform selectivity and supports a structural basis for the compound’s enhanced safety profile. Because the PDE4A10 model used for docking represents only a single enzyme conformation, future work will experimentally validate these predicted interactions through co-crystallization of the PDE4A10:11h complex and site-directed mutagenesis, to confirm the docking results and better define the mechanistic link between binding and tolerability.

The medicinal chemistry campaign that identified 11h reported its selectivity for PDE4 over other PDE families, with a modest preference for PDE4B and IC_50_ values in the micromolar range (Vadukoot et al., 2020; Burkovetskaya et al., 2020). Our findings refine this profile by showing potent inhibition across all four PDE4 isoforms, including two PDE4D splice variants, with low nanomolar IC_50_ values, while maintaining modest selectivity over PDE4A and PDE4C. These differences likely reflect methodological advances. For example, unlike the previously published study which used truncated catalytic domains (Mori et al., 2010), we employed full-length PDE4 constructs to preserve native regulatory dynamics that are relevant to physiological binding. Comparatively, 11h binds to PDE4 isoforms with potency similar to that of other selective PDE4 inhibitors, including rolipram and roflumilast (Jin et al., 2023).

11h’s pharmacological activity translated across multiple inflammatory models. *In vitro*, 11h dose-dependently suppressed the release of TNFα, IL-6, and nitrites in LPS-stimulated mouse macrophages, IMG microglia-like cells, and human PBMCs, without impairing cell viability. These anti-inflammatory effects were further validated in a murine model of LPS-induced neuroinflammation, where a single dose of 11h significantly decreased CD86^+^ (M1) macrophages and CD4^+^ T cells, while increasing CD206^+^ (M2) macrophages, key players in tissue repair and anti-inflammatory signaling. Cellular shifts were accompanied by reduced expression of pro-inflammatory cytokines and oxidative stress markers, suggesting that 11h reprograms the inflammatory microenvironment. Consistent with reports showing that PDE4 inhibitors can promote macrophage polarization from M1 to M2 states, these findings support 11h’s potential in treating CNS diseases with a prominent neuroinflammatory component (Kang et al., 2023; Song and Suk, 2017). Mechanistically, 11h also downregulated the expression of genes involved in the MyD88 signaling pathway, a central mediator of TLR4- and IL-1R-driven inflammatory cascades *via* NF-κB activation. Dysregulated MyD88 signaling contributes to the pathogenesis of numerous CNS diseases, including AD and MS (Chen H. et al., 2025; Zheng et al., 2019). Our data suggest that 11h may attenuate neuroinflammation by modulating MyD88-dependent pathways in microglia and other immune cells. Further studies are warranted to determine whether these effects extend to additional cell types (e.g., neurons, astrocytes) and to other pathways implicated in neuroinflammation (e.g., TRIF, Nrf2).

The pharmacokinetic profile of 11h was consistent with previous reports in mice and applicable to CNS targeting (Burkovetskaya et al., 2020). In rats, 11h demonstrated robust and dose-proportional systemic exposure, as well as potent brain penetration, with brain tissue levels exceeding plasma concentrations for up to 48 h post-dose. Importantly, brain exposure following oral administration was markedly higher than that reported for ibudilast, despite a five-fold lower dose (Sanftner et al., 2009). A secondary plasma concentration peak at 10 mg/kg suggests that 11h may undergo enterohepatic recirculation, which could explain its prolonged systemic exposure, as well as the differences observed in oral bioavailability between rats and mice (Roberts et al., 2002). Metabolite identification studies are currently in progress to elucidate how the biotransformation of 11h may impact its overall pharmacokinetic activity.

11h exhibits a strong safety profile across a range of preclinical assays. No genotoxicity or cytotoxicity was observed in AMES assays, and the compound exhibited minimal inhibition of cardiac ion channels, suggesting low risk for proarrhythmic events. 11h showed negligible off-target activity against a broad panel of CNS-relevant receptors, transporters, and ion channels, and exhibited minimal CYP450 inhibition, reducing the potential for drug-drug interactions. Behavioral studies further support the tolerability of 11h, as repeated dosing did not cause significant changes in weight, or induce anxiety- or depressive-like behaviors in rats. In fact, animals treated with 11h spent more time in the open arms of the EZM and showed reduced immobility in the FST, suggesting potential anxiolytic and antidepressant-like effects. These results are consistent with previous findings which highlight the therapeutic relevance of PDE4 modulation in mood-related disorders and warrant future investigations in translatable models of depression (Wang et al., 2024; Li et al., 2009). Importantly, unlike ibudilast, 11h did not induce vomiting in the ferret model even at more than 50-fold the therapeutic dose observed in rodent disease models. This safety profile was accompanied by robust suppression of plasma TNFα, an established pharmacodynamic marker of PDE4 inhibition (Wyska, 2010; Świerczek et al., 2017). Notably, 11h achieved comparable TNFα reduction to ibudilast at one-twentieth the dose, underscoring its improved tolerability and maintained efficacy.

Previous studies have demonstrated that 11h attenuates cocaine-mediated reward behaviors *in vivo*, suggesting therapeutic potential beyond its anti-neuroinflammatory effects (Burkovetskaya et al., 2020). When considered alongside the data presented here, these findings imply that 11h may also modulate neuronal excitation and inhibition through its ability to elevate intracellular cAMP levels. Similar neuromodulatory effects have been observed with other PDE4 inhibitors, providing precedent for the broad therapeutic utility of this drug class across both neuroinflammatory and neuropsychiatric indications (Liu et al., 2017). Considering this, current studies are ongoing to further explore the potential pro-cognitive effects of 11h across various neuropsychiatric and neurodegenerative disease models.

While these findings strongly support the continued investigation of 11h as a therapeutic candidate for CNS disorders, several limitations in these studies should be noted. First, all *in vivo* studies were conducted exclusively in male animals, precluding the assessment of sex-specific effects. This represents a significant gap, as many neurological diseases, including migraine, MS, AD, and depression, disproportionately affect women. Future studies incorporating both sexes will be critical for evaluating potential sex differences in the pharmacological response to 11h. Additionally, variations in dosing regimens and routes of administration across experiments within the same species reflected the exploratory nature of this early-stage work. Despite this, our pharmacokinetic and efficacy data suggest that 11h is well suited for oral administration, and further studies are underway to evaluate optimal oral dosing strategies in disease-relevant models. Finally, the MTD of 11h was not reached in these studies, limiting our ability to fully define the compound’s therapeutic window. Future dose-escalation and toxicology studies are needed to establish the MTD and inform the design of subsequent safety and efficacy assessments.

## Conclusion

11h emerges as a differentiated next-generation PDE4 inhibitor with the potential to overcome historical barriers to clinical adoption. By coupling broad PDE4 inhibition with favorable pharmacokinetics and CNS penetration, while maintaining a well-tolerated safety profile, 11h positions itself as a viable therapeutic candidate where earlier agents failed. Its dose proportionality and suitability for repeat dosing further strengthen its translational potential. Most notably, 11h achieves anti-inflammatory efficacy without inducing emesis or behavioral toxicity, providing a compelling rationale for its advancement into clinical development.

## Data Availability

The original contributions presented in the study are included in the article/Supplementary Material, further inquiries can be directed to the corresponding author.

## References

[B1] Armstrong P. Güngör H. Anongjanya P. Tweedy C. Parkin E. Johnston J. (2024). Protective effect of PDE4B subtype-specific inhibition in an App knock-in mouse model for Alzheimer's disease. Neuropsychopharmacology 49 (10), 1559–1568. 10.1038/s41386-024-01852-z 38521860 PMC11319650

[B2] Banerjee A. Lu Y. Do K. Mize T. Wu X. Chen X. (2021). Validation of induced microglia-like cells (iMG cells) for future studies of brain diseases. Front. Cell Neurosci. 15, 629279. 10.3389/fncel.2021.629279 33897370 PMC8063054

[B3] Barnette M. S. Grous M. Cieslinski L. B. Burman M. Christensen S. B. Torphy T. J. (1995). Inhibitors of phosphodiesterase IV (PDE IV) increase acid secretion in rabbit isolated gastric glands: correlation between function and interaction with a high-affinity rolipram binding site. J. Pharmacol. Exp. Ther. 273 (3), 1396–1402. 10.1016/s0022-3565(25)09664-8 7791113

[B4] Blokland A. Heckman P. Vanmierlo T. Schreiber R. Paes D. Prickaerts J. (2019a). Phosphodiesterase type 4 inhibition in CNS diseases. Trends Pharmacol. Sci. 40 (12), 971–985. 10.1016/j.tips.2019.10.006 31704172

[B5] Blokland A. Van Duinen M. A. Sambeth A. Heckman P. R. A. Tsai M. Lahu G. (2019b). Acute treatment with the PDE4 inhibitor roflumilast improves verbal word memory in healthy old individuals: a double-blind placebo-controlled study. Neurobiol. Aging 77, 37–43. 10.1016/j.neurobiolaging.2019.01.014 30776650

[B6] Bollen E. Puzzo D. Rutten K. Privitera L. De Vry J. Vanmierlo T. (2014). Improved long-term memory via enhancing cGMP-PKG signaling requires cAMP-PKA signaling. Neuropsychopharmacology 39 (11), 2497–2505. 10.1038/npp.2014.106 24813825 PMC4207334

[B7] Boyd A. Aragon I. V. Rich J. McDonough W. Oditt M. Irelan D. (2021). Assessment of PDE4 inhibitor-induced hypothermia as a correlate of nausea in mice. Biology 10 (12), 1355. 10.3390/biology10121355 34943270 PMC8698290

[B8] Braun A. A. Skelton M. R. Vorhees C. V. Williams M. T. (2011). Comparison of the elevated plus and elevated zero mazes in treated and untreated male Sprague-Dawley rats: effects of anxiolytic and anxiogenic agents. Pharmacol. Biochem. Behav. 97 (3), 406–415. 10.1016/j.pbb.2010.09.013 20869983 PMC3006066

[B9] Brideau C. Van Staden C. Styhler A. Rodger I. W. Chan C. C. (1999). The effects of phosphodiesterase type 4 inhibitors on tumour necrosis factor-alpha and leukotriene B4 in a novel human whole blood assay. Br. J. Pharmacol. 126 (4), 979–988. 10.1038/sj.bjp.0702387 10193778 PMC1571215

[B10] Burgin A. B. Magnusson O. T. Singh J. Witte P. Staker B. L. Bjornsson J. M. (2010). Design of phosphodiesterase 4D (PDE4D) allosteric modulators for enhancing cognition with improved safety. Nat. Biotechnol. 28 (1), 63–70. 10.1038/nbt.1598 20037581

[B11] Burkovetskaya M. E. Liu Q. Vadukoot A. K. Gautam N. Alnouti Y. Kumar S. (2020). KVA-D-88, a novel preferable phosphodiesterase 4B inhibitor, decreases cocaine-mediated reward properties in *vivo* . ACS Chem. Neurosci. 11 (15), 2231–2242. 10.1021/acschemneuro.0c00170 32609488 PMC8383802

[B12] Chen J. C. Chen P. C. Chiang Y. C. (2009). Molecular mechanisms of psychostimulant addiction. Chang. Gung Med. J. 32 (2), 148–154. 19403004

[B13] Chen H. Chang X. Zhou J. Zhang G. Cheng J. Zhang Z. (2025). Anti-neuroinflammatory and neuroprotective effects of T-006 on Alzheimer's Disease models by modulating TLR4-Mediated MyD88/NF-κB signaling. CNS Neurol. Disord. Drug Targets 24, 382–396. 10.2174/0118715273337232241121113048 39791155

[B14] Chen Y. Kou Y. Ni Y. Yang H. Xu C. Fan H. (2025). Microglia efferocytosis: an emerging mechanism for the resolution of neuroinflammation in Alzheimer's disease. J. Neuroinflammation 22 (1), 96. 10.1186/s12974-025-03428-0 40159486 PMC11955113

[B15] Cong Y. F. Liu F. W. Xu L. Song S. S. Shen X. R. Liu D. (2023). Rolipram ameliorates memory deficits and depression-like behavior in APP/PS1/tau triple transgenic mice: involvement of neuroinflammation and apoptosis via cAMP signaling. Int. J. Neuropsychopharmacol. 26 (9), 585–598. 10.1093/ijnp/pyad042 37490542 PMC10519811

[B16] Conti M. Richter W. Mehats C. Livera G. Park J. Y. Jin C. (2003). Cyclic AMP-specific PDE4 phosphodiesterases as critical components of cyclic AMP signaling. J. Biol. Chem. 278 (8), 5493–5496. 10.1074/jbc.R200029200 12493749

[B17] Crespi C. L. Miller V. P. Penman B. W. (1997). Microtiter plate assays for inhibition of human, drug-metabolizing cytochromes P450. Anal. Biochem. 248 (1), 188–190. 10.1006/abio.1997.2145 9177742

[B18] Donders Z. Skorupska I. J. Willems E. Mussen F. Broeckhoven J. V. Carlier A. (2024). Beyond PDE4 inhibition: a comprehensive review on downstream cAMP signaling in the central nervous system. Biomed. & Pharmacother. 177, 117009. 10.1016/j.biopha.2024.117009 38908196

[B19] Duplantier A. J. Biggers M. S. Chambers R. J. Cheng J. B. Cooper K. Damon D. B. (1996). Biarylcarboxylic acids and -amides: inhibition of phosphodiesterase type IV versus [3H]rolipram binding activity and their relationship to emetic behavior in the ferret. J. Med. Chem. 39 (1), 120–125. 10.1021/jm9505066 8568798

[B20] Ernst O. Glucksam-Galnoy Y. Athamna M. Ben-Dror I. Ben-Arosh H. Levy-Rimler G. (2019). The cAMP pathway amplifies early MyD88-Dependent and type I interferon-independent LPS-induced Interleukin-10 expression in mouse macrophages. Mediat. Inflamm. 2019, 3451461. 10.1155/2019/3451461 31148944 PMC6501241

[B21] Fox R. J. Coffey C. S. Conwit R. Cudkowicz M. E. Gleason T. Goodman A. (2018). Phase 2 trial of ibudilast in progressive multiple sclerosis. N. Engl. J. Med. 379 (9), 846–855. 10.1056/NEJMoa1803583 30157388 PMC6172944

[B22] Gardner C. J. Twissell D. J. Dale T. J. Gale J. D. Jordan C. C. Kilpatrick G. J. (1995). The broad-spectrum anti-emetic activity of the novel non-peptide tachykinin NK1 receptor antagonist GR203040. Br. J. Pharmacol. 116 (8), 3158–3163. 10.1111/j.1476-5381.1995.tb15118.x 8719790 PMC1909155

[B23] Gilleen J. Farah Y. Davison C. Kerins S. Valdearenas L. Uz T. (2021). An experimental medicine study of the phosphodiesterase-4 inhibitor, roflumilast, on working memory-related brain activity and episodic memory in schizophrenia patients. Psychopharmacol. Berl. 238 (5), 1279–1289. 10.1007/s00213-018-5134-y 30536081 PMC8062361

[B24] Gong B. Vitolo O. V. Trinchese F. Liu S. Shelanski M. Arancio O. (2004). Persistent improvement in synaptic and cognitive functions in an Alzheimer mouse model after rolipram treatment. J. Clin. Invest. 114 (11), 1624–1634. 10.1172/JCI22831 15578094 PMC529285

[B25] Holbrook M. Coker S. J. (1991). Effects of zaprinast and rolipram on platelet aggregation and arrhythmias following myocardial ischaemia and reperfusion in anaesthetized rabbits. Br. J. Pharmacol. 103 (4), 1973–1979. 10.1111/j.1476-5381.1991.tb12362.x 1655149 PMC1908205

[B26] Imanishi T. Sawa A. Ichimaru Y. Miyashiro M. Kato S. Yamamoto T. (1997). Ameliorating effects of rolipram on experimentally induced impairments of learning and memory in rodents. Eur. J. Pharmacol. 321 (3), 273–278. 10.1016/s0014-2999(96)00969-7 9085037

[B27] Jacobitz S. McLaughlin M. M. Livi G. P. Burman M. Torphy T. J. (1996). Mapping the functional domains of human recombinant phosphodiesterase 4A: structural requirements for catalytic activity and rolipram binding. Mol. Pharmacol. 50 (4), 891–899. 10.1016/s0026-895x(25)09391-5 8863835

[B28] Janes A. C. Kantak K. M. Cherry J. A. (2009). The involvement of type IV phosphodiesterases in cocaine-induced sensitization and subsequent pERK expression in the mouse nucleus accumbens. Psychopharmacol. Berl. 206 (2), 177–185. 10.1007/s00213-009-1594-4 19588125

[B29] Jin S. L. Conti M. (2002). Induction of the cyclic nucleotide phosphodiesterase PDE4B is essential for LPS-activated TNF-alpha responses. Proc. Natl. Acad. Sci. U. S. A. 99 (11), 7628–7633. 10.1073/pnas.122041599 12032334 PMC124305

[B30] Jin S.-L. C. Lan L. Zoudilova M. Conti M. (2005). Specific role of phosphodiesterase 4B in lipopolysaccharide-induced signaling in mouse Macrophages1. J. Immunol. 175 (3), 1523–1531. 10.4049/jimmunol.175.3.1523 16034090

[B31] Jin J. Mazzacuva F. Crocetti L. Giovannoni M. P. Cilibrizzi A. (2023). PDE4 inhibitors: profiling hits through the multitude of structural classes. Int. J. Mol. Sci. 24 (14), 11518. 10.3390/ijms241411518 37511275 PMC10380597

[B32] Kandel E. R. (2012). The molecular biology of memory: cAMP, PKA, CRE, CREB-1, CREB-2, and CPEB. Mol. Brain 5 (1), 14. 10.1186/1756-6606-5-14 22583753 PMC3514210

[B33] Kang D. H. Ahn S. Chae J. W. Song J. S. (2023). Differential effects of two phosphodiesterase 4 inhibitors against lipopolysaccharide-induced neuroinflammation in mice. BMC Neurosci. 24 (1), 39. 10.1186/s12868-023-00810-7 37525115 PMC10391911

[B34] Krause W. Kühne G. (1988). Pharmacokinetics of rolipram in the rhesus and cynomolgus monkeys, the rat and the rabbit. Studies on species differences. Xenobiotica 18 (5), 561–571. 10.3109/00498258809041693 3400274

[B35] Lakics V. Karran E. H. Boess F. G. (2010). Quantitative comparison of phosphodiesterase mRNA distribution in human brain and peripheral tissues. Neuropharmacology 59 (6), 367–374. 10.1016/j.neuropharm.2010.05.004 20493887

[B36] Li Y. F. Huang Y. Amsdell S. L. Xiao L. O'Donnell J. M. Zhang H. T. (2009). Antidepressant- and anxiolytic-like effects of the phosphodiesterase-4 inhibitor rolipram on behavior depend on cyclic AMP response element binding protein-mediated neurogenesis in the hippocampus. Neuropsychopharmacology 34 (11), 2404–2419. 10.1038/npp.2009.66 19516250 PMC2743762

[B37] Li H. Zuo J. Tang W. (2018). Phosphodiesterase-4 inhibitors for the treatment of inflammatory diseases. Front. Pharmacol. 9, 1048. 10.3389/fphar.2018.01048 30386231 PMC6199465

[B38] Liu X. Zhong P. Vickstrom C. Li Y. Liu Q. S. (2017). PDE4 inhibition restores the balance between excitation and inhibition in VTA dopamine neurons disrupted by repeated *in vivo* cocaine exposure. Neuropsychopharmacology 42 (10), 1991–1999. 10.1038/npp.2017.96 28497801 PMC5561351

[B39] Maron D. M. Ames B. N. (1983). Revised methods for the Salmonella mutagenicity test. Mutat. Res. 113 (3-4), 173–215. 10.1016/0165-1161(83)90010-9 6341825

[B40] McDonough W. Aragon I. V. Rich J. Murphy J. M. Abou Saleh L. Boyd A. (2020). PAN-selective inhibition of cAMP-phosphodiesterase 4 (PDE4) induces gastroparesis in mice. Faseb J. 34 (9), 12533–12548. 10.1096/fj.202001016RR 32738081 PMC7954513

[B41] Miró X. Pérez-Torres S. Puigdomènech P. Palacios J. M. Mengod G. (2002). Differential distribution of PDE4D splice variant mRNAs in rat brain suggests association with specific pathways and presynaptical localization. Synapse 45 (4), 259–269. 10.1002/syn.10100 12125047

[B42] Moreira F. A. Crippa J. A. (2009). The psychiatric side-effects of rimonabant. Braz J. Psychiatry 31 (2), 145–153. 10.1590/s1516-44462009000200012 19578688

[B43] Mori F. Pérez-Torres S. De Caro R. Porzionato A. Macchi V. Beleta J. (2010). The human area postrema and other nuclei related to the emetic reflex express cAMP phosphodiesterases 4B and 4D. J. Chem. Neuroanat. 40 (1), 36–42. 10.1016/j.jchemneu.2010.03.004 20347962

[B44] Naganuma K. Omura A. Maekawara N. Saitoh M. Ohkawa N. Kubota T. (2009). Discovery of selective PDE4B inhibitors. Bioorg Med. Chem. Lett. 19 (12), 3174–3176. 10.1016/j.bmcl.2009.04.121 19447034

[B45] Paes D. Schepers M. Willems E. Rombaut B. Tiane A. Solomina Y. (2023). Ablation of specific long PDE4D isoforms increases neurite elongation and conveys protection against amyloid-β pathology. Cell Mol. Life Sci. 80 (7), 178. 10.1007/s00018-023-04804-w 37306762 PMC10261250

[B46] Pearse D. D. Hughes Z. A. (2016). PDE4B as a microglia target to reduce neuroinflammation. Glia 64 (10), 1698–1709. 10.1002/glia.22986 27038323

[B47] Percie du Sert N. Hurst V. Ahluwalia A. Alam S. Avey M. T. Baker M. (2020). The ARRIVE guidelines 2.0: updated guidelines for reporting animal research. PLoS Biol. 18 (7), e3000410. 10.1371/journal.pbio.3000410 32663219 PMC7360023

[B48] Pérez-Torres S. Miró X. Palacios J. M. Cortés R. Puigdoménech P. Mengod G. (2000). Phosphodiesterase type 4 isozymes expression in human brain examined by *in situ* hybridization histochemistry and[3H]rolipram binding autoradiography. Comparison with monkey and rat brain. J. Chem. Neuroanat. 20 (3-4), 349–374. 10.1016/s0891-0618(00)00097-1 11207431

[B49] Roberts M. S. Magnusson B. M. Burczynski F. J. Weiss M. (2002). Enterohepatic circulation: physiological, pharmacokinetic and clinical implications. Clin. Pharmacokinet. 41 (10), 751–790. 10.2165/00003088-200241100-00005 12162761

[B50] Robichaud A. Tattersall F. D. Choudhury I. Rodger I. W. (1999). Emesis induced by inhibitors of type IV cyclic nucleotide phosphodiesterase (PDE IV) in the ferret. Neuropharmacology 38 (2), 289–297. 10.1016/s0028-3908(98)00190-7 10218871

[B51] Rocque W. J. Tian G. Wiseman J. S. Holmes W. D. Zajac-Thompson I. Willard D. H. (1997). Human recombinant phosphodiesterase 4B2B binds (R)-rolipram at a single site with two affinities. Biochemistry 36 (46), 14250–14261. 10.1021/bi971112e 9369498

[B52] Rombaut B. Kessels S. Schepers M. Tiane A. Paes D. Solomina Y. (2021). PDE inhibition in distinct cell types to reclaim the balance of synaptic plasticity. Theranostics 11 (5), 2080–2097. 10.7150/thno.50701 33500712 PMC7797685

[B53] Russo A. Patanè G. T. Putaggio S. Lombardo G. E. Ficarra S. Barreca D. (2024). Mechanisms underlying the effects of chloroquine on red blood cells metabolism. Int. J. Mol. Sci. 25 (12), 6424. 10.3390/ijms25126424 38928131 PMC11203553

[B54] Sanftner L. M. Gibbons J. A. Gross M. I. Suzuki B. M. Gaeta F. C. Johnson K. W. (2009). Cross-species comparisons of the pharmacokinetics of ibudilast. Xenobiotica 39 (12), 964–977. 10.3109/00498250903254340 19925385

[B55] Schepers M. Paes D. Tiane A. Rombaut B. Piccart E. van Veggel L. (2023). Selective PDE4 subtype inhibition provides new opportunities to intervene in neuroinflammatory *versus* myelin damaging hallmarks of multiple sclerosis. Brain, Behav. Immun. 109, 1–22. 10.1016/j.bbi.2022.12.020 36584795

[B56] Singh D. (2022). Astrocytic and microglial cells as the modulators of neuroinflammation in Alzheimer's disease. J. Neuroinflammation 19 (1), 206. 10.1186/s12974-022-02565-0 35978311 PMC9382837

[B57] Slattery D. A. Cryan J. F. (2012). Using the rat forced swim test to assess antidepressant-like activity in rodents. Nat. Protoc. 7 (6), 1009–1014. 10.1038/nprot.2012.044 22555240

[B58] Song G. J. Suk K. (2017). Pharmacological modulation of functional phenotypes of microglia in neurodegenerative diseases. Front. Aging Neurosci. 9, 139. 10.3389/fnagi.2017.00139 28555105 PMC5430023

[B59] Souness J. E. Rao S. (1997). Proposal for pharmacologically distinct conformers of PDE4 cyclic AMP phosphodiesterases. Cell Signal 9 (3-4), 227–236. 10.1016/s0898-6568(96)00173-8 9218122

[B60] Sumbria R. K. Grigoryan M. M. Vasilevko V. Krasieva T. B. Scadeng M. Dvornikova A. K. (2016). A murine model of inflammation-induced cerebral microbleeds. J. Neuroinflammation 13 (1), 218. 10.1186/s12974-016-0693-5 27577728 PMC5006574

[B61] Świerczek A. Wyska E. Baś S. Woyciechowska M. Mlynarski J. (2017). PK/PD studies on non-selective PDE inhibitors in rats using cAMP as a marker of pharmacological response. Naunyn Schmiedeb. Arch. Pharmacol. 390 (10), 1047–1059. 10.1007/s00210-017-1406-z 28730281 PMC5599463

[B62] Taciak B. Białasek M. Braniewska A. Sas Z. Sawicka P. Kiraga Ł. (2018). Evaluation of phenotypic and functional stability of RAW 264.7 cell line through serial passages. PLoS One 13 (6), e0198943. 10.1371/journal.pone.0198943 29889899 PMC5995401

[B63] Titus D. J. Wilson N. M. Freund J. E. Carballosa M. M. Sikah K. E. Furones C. (2016). Chronic cognitive dysfunction after traumatic brain injury is improved with a phosphodiesterase 4B inhibitor. J. Neurosci. 36 (27), 7095–7108. 10.1523/JNEUROSCI.3212-15.2016 27383587 PMC4938858

[B64] Vadukoot A. K. Sharma S. Aretz C. D. Kumar S. Gautam N. Alnouti Y. (2020). Synthesis and SAR studies of 1H-Pyrrolo[2,3-b]pyridine-2-carboxamides as phosphodiesterase 4B (PDE4B) inhibitors. ACS Med. Chem. Lett. 11 (10), 1848–1854. 10.1021/acsmedchemlett.9b00369 33062163 PMC7549101

[B65] Vetreno R. P. Qin L. Coleman L. G. Jr. Crews F. T. (2021). Increased toll-like Receptor-MyD88-NFκB-Proinflammatory neuroimmune signaling in the orbitofrontal cortex of humans with alcohol use disorder. Alcohol Clin. Exp. Res. 45 (9), 1747–1761. 10.1111/acer.14669 34415075 PMC8526379

[B66] Wachtel H. Schneider H. H. (1986). Rolipram, a novel antidepressant drug, reverses the hypothermia and hypokinesia of monoamine-depleted mice by an action beyond postsynaptic monoamine receptors. Neuropharmacology 25 (10), 1119–1126. 10.1016/0028-3908(86)90159-0 2946976

[B67] Walf A. A. Frye C. A. (2007). The use of the elevated plus maze as an assay of anxiety-related behavior in rodents. Nat. Protoc. 2 (2), 322–328. 10.1038/nprot.2007.44 17406592 PMC3623971

[B68] Wang Z. Zhang Y. Chai J. Wu Y. Zhang W. Zhang Z. (2024). Roflumilast: modulating neuroinflammation and improving motor function and depressive symptoms in multiple sclerosis. J. Affect Disord. 350, 761–773. 10.1016/j.jad.2023.12.074 38220100

[B69] Wyska E. (2010). Pharmacokinetic-pharmacodynamic modeling of methylxanthine derivatives in mice challenged with high-dose lipopolysaccharide. Pharmacology 85 (5), 264–271. 10.1159/000288734 20389149

[B70] Zeller E. Stief H. J. Pflug B. Sastre-y-Hernández M. (1984). Results of a phase II study of the antidepressant effect of rolipram. Pharmacopsychiatry 17 (6), 188–190. 10.1055/s-2007-1017435 6393150

[B71] Zhang C. Xu Y. Zhang H. T. Gurney M. E. O'Donnell J. M. (2017). Comparison of the pharmacological profiles of selective PDE4B and PDE4D inhibitors in the central nervous system. Sci. Rep. 7, 40115. 10.1038/srep40115 28054669 PMC5215650

[B72] Zhang F. F. Wang H. Zhou Y. M. Yu H. Y. Zhang M. Du X. (2022). Inhibition of phosphodiesterase-4 in the spinal dorsal horn ameliorates neuropathic pain via cAMP-cytokine-Cx43 signaling in mice. CNS Neurosci. Ther. 28 (5), 749–760. 10.1111/cns.13807 35156776 PMC8981432

[B73] Zheng C. Chen J. Chu F. Zhu J. Jin T. (2019). Inflammatory role of TLR-MyD88 signaling in multiple sclerosis. Front. Mol. Neurosci. 12, 314. 10.3389/fnmol.2019.00314 31998072 PMC6965019

