# Supplementary material for: Pharmacological characterization and preclinical evaluation of 11h: a novel, brain-penetrant PDE4 inhibitor for neurological disorders
Source: Front Pharmacol. 2026 Jan 8;16:1720327. doi: 10.3389/fphar.2025.1720327 (PMC12823826; doi:10.3389/fphar.2025.1720327)
Supplement: Supplementary file 1 [file DataSheet1.pdf]

Supplementary Material

Supplementary Table 1. Mutagenicity assessment of 11h using the bacterial reverse mutation (AMES) assay. 11h was tested at concentrations of 5.0 to 100 μM across four tester strains (TA98, TA100, TA1535, and TA1537) in the presence and absence of metabolic activation (rat-liver S9 fraction). Concurrent bacterial cytotoxicity assays were performed to verify assay integrity and aid in interpretation of mutagenicity results. A "–" indicates no mutagenic response detected, while a "+" denotes a weakly positive response ($p < 0.05$).

Strains	TA98			TA100			TA1535			TA1537		
Experiments	Cytotox	S9 -	S9 +	Cytotox	S9 -	S9 +	Cytotox	S9 -	S9 +	Cytotox	S9 -	S9 +
1st	-	-	-	-	-	-	-	-	-	-	-	+
2nd	-	-	-	-	-	-	-	-	-	-	-	+

Supplementary Table 2. Blood cell partitioning of 11h across multiple species. The distribution of 11h between red blood cells and plasma was determined using a standard blood cell partitioning assay and compared to the highly lipophilic reference compound, chloroquine. The blood-to-plasma partition coefficient ($K_{b/p}$) was calculated for each species. Across all species, 11h exhibited consistently low $K_{b/p}$ values relative to chloroquine, indicating limited accumulation in red blood cells. All experiments were performed in duplicate.

**Blood to Plasma Ratio ($K_{b/p}$)**		
	**11h**	**Chloroquine**
Human	0.80	2.51
Dog	0.68	3.22
Rat	0.86	2.00
Mouse	0.73	2.06

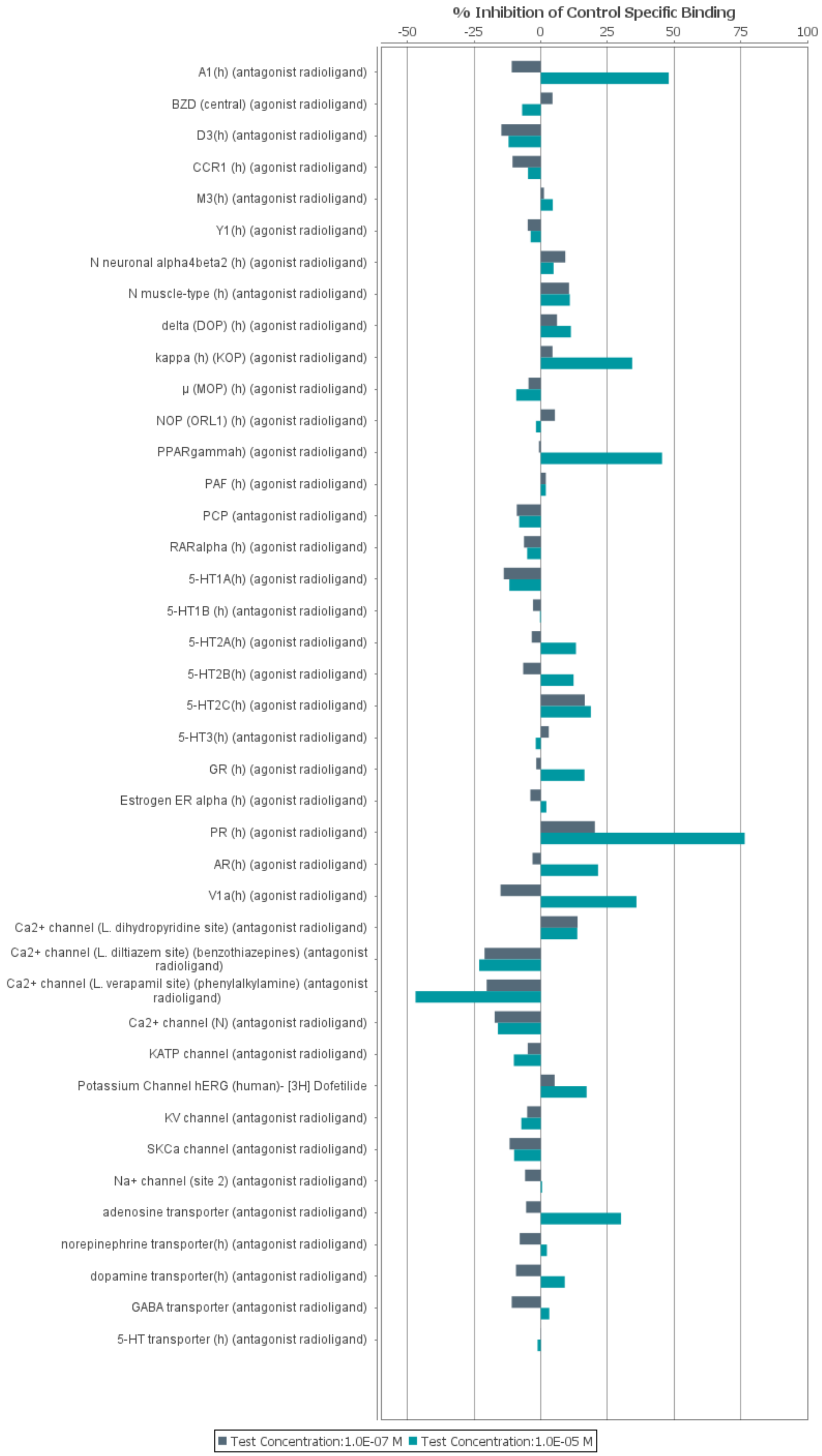
% Inhibition of Control Specific Binding
-50
-25
0
25
50
75
100
A1(h) (antagonist radioligand)
BZD (central) (agonist radioligand)
D3(h) (antagonist radioligand)
CCR1 (h) (agonist radioligand)
M3(h) (antagonist radioligand)
Y1(h) (agonist radioligand)
N neuronal alpha4beta2 (h) (agonist radioligand)
N muscle-type (h) (antagonist radioligand)
delta (DOP) (h) (agonist radioligand)
kappa (h) (KOP) (agonist radioligand)
μ (MOP) (h) (agonist radioligand)
NOP (ORL1) (h) (agonist radioligand)
PPARgammah) (agonist radioligand)
PAF (h) (agonist radioligand)
PCP (antagonist radioligand)
RARalpha (h) (agonist radioligand)
5-HT1A(h) (agonist radioligand)
5-HT1B (h) (antagonist radioligand)
5-HT2A(h) (agonist radioligand)
5-HT2B(h) (agonist radioligand)
5-HT2C(h) (agonist radioligand)
5-HT3(h) (antagonist radioligand)
GR (h) (agonist radioligand)
Estrogen ER alpha (h) (agonist radioligand)
PR (h) (agonist radioligand)
AR(h) (agonist radioligand)
V1a(h) (agonist radioligand)
Ca2+ channel (L. dihydropyridine site) (antagonist radioligand)
Ca2+ channel (L. diltiazem site) (benzothiazepines) (antagonist radioligand)
Ca2+ channel (L. verapamil site) (phenylalkylamine) (antagonist radioligand)
Ca2+ channel (N) (antagonist radioligand)
KATP channel (antagonist radioligand)
Potassium Channel hERG (human)- [3H] Dofetilide
KV channel (antagonist radioligand)
SKCa channel (antagonist radioligand)
Na+ channel (site 2) (antagonist radioligand)
adenosine transporter (antagonist radioligand)
norepinephrine transporter(h) (antagonist radioligand)
dopamine transporter(h) (antagonist radioligand)
GABA transporter (antagonist radioligand)
5-HT transporter (h) (antagonist radioligand)
Test Concentration:1.0E-07 M
Test Concentration:1.0E-05 M

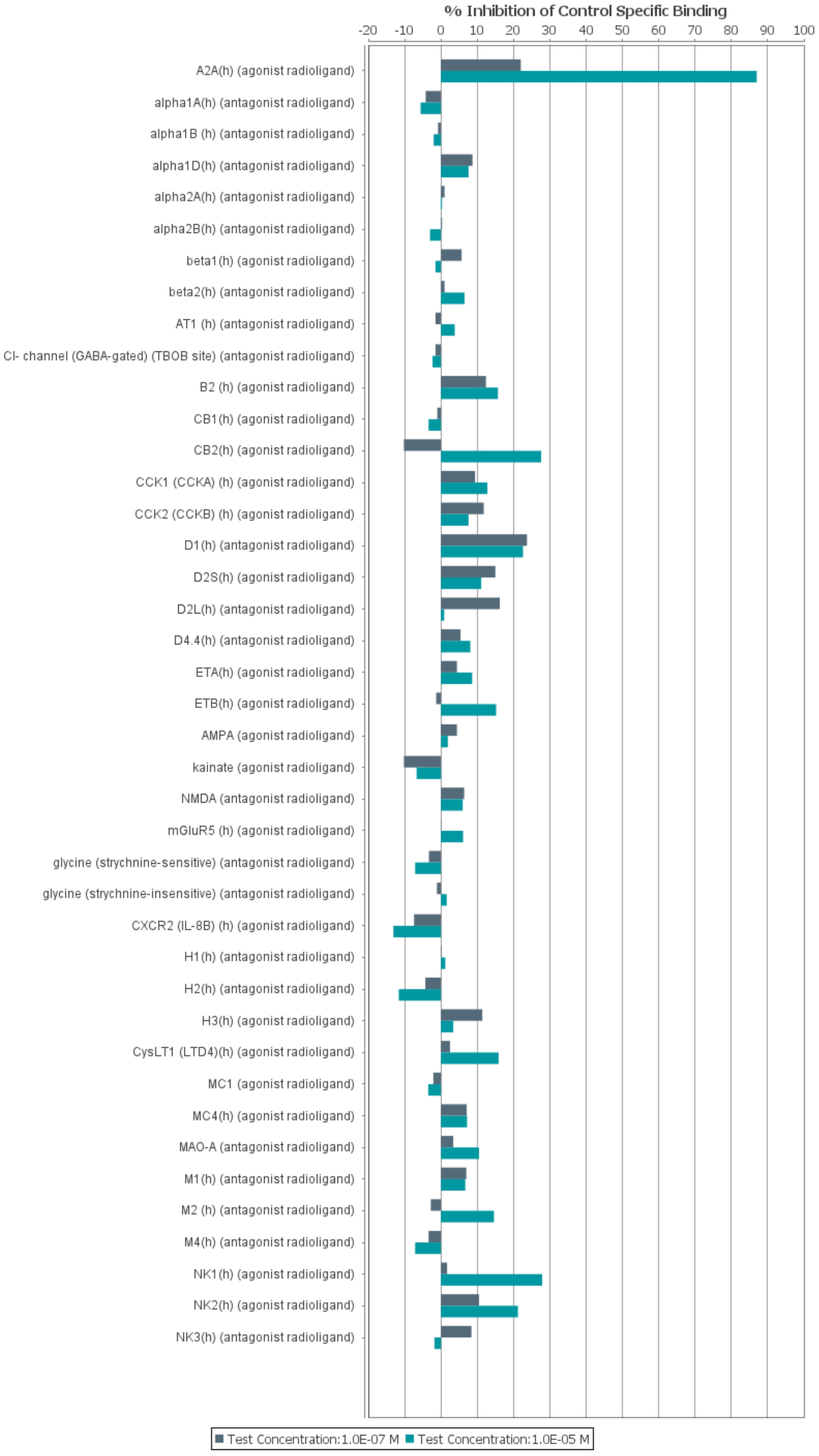
% Inhibition of Control Specific Binding
-20 -10 0 10 20 30 40 50 60 70 80 90 100
A2A(h) (agonist radioligand)
alpha1A(h) (antagonist radioligand)
alpha1B (h) (antagonist radioligand)
alpha1D(h) (antagonist radioligand)
alpha2A(h) (antagonist radioligand)
alpha2B(h) (antagonist radioligand)
beta1(h) (agonist radioligand)
beta2(h) (antagonist radioligand)
AT1 (h) (antagonist radioligand)
Cl- channel (GABA-gated) (TBOB site) (antagonist radioligand)
B2 (h) (agonist radioligand)
CB1(h) (agonist radioligand)
CB2(h) (agonist radioligand)
CCK1 (CCKA) (h) (agonist radioligand)
CCK2 (CCKB) (h) (agonist radioligand)
D1(h) (antagonist radioligand)
D2S(h) (agonist radioligand)
D2L(h) (antagonist radioligand)
D4.4(h) (antagonist radioligand)
ETA(h) (agonist radioligand)
ETB(h) (agonist radioligand)
AMPA (agonist radioligand)
kainate (agonist radioligand)
NMDA (antagonist radioligand)
mGluR5 (h) (agonist radioligand)
glycine (strychnine-sensitive) (antagonist radioligand)
glycine (strychnine-insensitive) (antagonist radioligand)
CXCR2 (IL-8B) (h) (agonist radioligand)
H1(h) (antagonist radioligand)
H2(h) (antagonist radioligand)
H3(h) (agonist radioligand)
CysLT1 (LTD4)(h) (agonist radioligand)
MC1 (agonist radioligand)
MC4(h) (agonist radioligand)
MAO-A (antagonist radioligand)
M1(h) (antagonist radioligand)
M2 (h) (antagonist radioligand)
M4(h) (antagonist radioligand)
NK1(h) (agonist radioligand)
NK2(h) (agonist radioligand)
NK3(h) (antagonist radioligand)
Test Concentration:1.0E-07 M
Test Concentration:1.0E-05 M

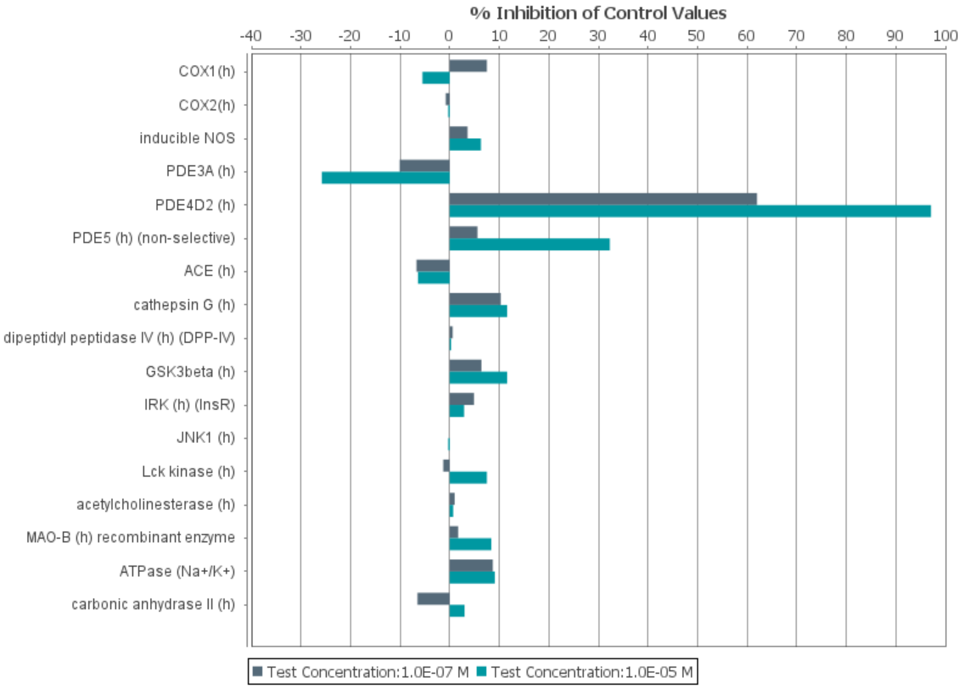

Supplementary Figure 1. Off-target binding profile of 11h in *in vitro* pharmacology assays. The binding activity of 11h was evaluated at 100 nM and 10 µM across a broad panel of receptors and enzymes to evaluate potential off-target interactions. 11h showed no significant binding to any target, with only weak affinity for the adenosine A_2A receptor (A_2A) and progesterone receptor (PR), both with IC_{50} values >10 µM. All assays were performed in duplicate.
